# New insights into the resistance mechanism for the BceAB-type transporter *Sa*NsrFP

**DOI:** 10.1038/s41598-022-08095-2

**Published:** 2022-03-10

**Authors:** Julia Gottstein, Julia Zaschke-Kriesche, Sandra Unsleber, Irina Voitsekhovskaia, Andreas Kulik, Lara V. Behrmann, Nina Overbeck, Kai Stühler, Evi Stegmann, Sander H. J. Smits

**Affiliations:** 1 Institute of Biochemistry, Heinrich-Heine-University Duesseldorf, Universitaetsstrasse 1, 40225 Duesseldorf, Germany; 2 Interfaculty Institute of Microbiology and Infection Medicin, Eberhard Karls University, Auf der Morgenstelle 28, 72076 Tübingen, Germany; 3 Molecular Proteomics Laboratory, Heinrich-Heine-University Duesseldorf, Universitaetsstrasse 1, 40225 Duesseldorf, Germany

**Keywords:** Antimicrobial resistance, Antimicrobials, Bacteria, Cellular microbiology

## Abstract

Treatment of bacterial infections is one of the major challenges of our time due to the evolved resistance mechanisms of pathogens against antibiotics. To circumvent this problem, it is necessary to understand the mode of action of the drug and the mechanism of resistance of the pathogen. One of the most potent antibiotic targets is peptidoglycan (PGN) biosynthesis, as this is an exclusively occurring and critical feature of bacteria. Lipid II is an essential PGN precursor synthesized in the cytosol and flipped into the outer leaflet of the membrane prior to its incorporation into nascent PGN. Antimicrobial peptides (AMPs), such as nisin and colistin, targeting PGN synthesis are considered promising weapons against multidrug-resistant bacteria. However, human pathogenic bacteria that were also resistant to these compounds evolved by the expression of an ATP-binding cassette transporter of the bacitracin efflux (BceAB) type localized in the membrane. In the human pathogen *Streptococcus agalactiae*, the BceAB transporter *Sa*NsrFP is known to confer resistance to the antimicrobial peptide nisin. The exact mechanism of action for *Sa*NsrFP is poorly understood. For a detailed characterization of the resistance mechanism, we heterologously expressed *Sa*NsrFP in *Lactococcus lactis*. We demonstrated that *Sa*NsrFP conferred resistance not only to nisin but also to a structurally diverse group of antimicrobial PGN-targeting compounds such as ramoplanin, lysobactin, or bacitracin/(Zn)-bacitracin. Growth experiments revealed that *Sa*NsrFP-producing cells exhibited normal behavior when treated with nisin and/or bacitracin, in contrast to the nonproducing cells, for which growth was significantly reduced. We further detected the accumulation of PGN precursors in the cytoplasm after treating the cells with bacitracin. This did not appear when *Sa*NsrFP was produced. Whole-cell proteomic protein experiments verified that the presence of *Sa*NsrFP in *L. lactis* resulted in higher production of several proteins associated with cell wall modification. These included, for example, the *N*-acetylmuramic acid-6-phosphate etherase MurQ and UDP-glucose 4-epimerase. Analysis of components of the cell wall of *Sa*NsrFP-producing cells implied that the transporter is involved in cell wall modification. Since we used an ATP-deficient mutant of the transporter as a comparison, we can show that *Sa*NsrFP and its inactive mutant do not show the same phenotype, albeit expressed at similar levels, which demonstrates the ATP dependency of the mediated resistance processes. Taken together, our data agree to a target protection mechanism and imply a direct involvement of *Sa*NsrFP in resistance by shielding the membrane-localized target of these antimicrobial peptides, resulting in modification of the cell wall.

## Introduction

Bacterial infections cause over 150,000 deaths every year and are a major threat for humans^1,2^. The treatment of many infectious diseases is possible due to the development of antibiotics, which have been discovered over the last 100 years, starting with penicillin in 1929. In recent years, however, antibiotic resistance has become a major challenge, as pathogenic bacteria have evolved several resistance mechanisms against antibiotics in use^3^.

An Achilles heel of bacteria is the synthesis pathway of peptidoglycan (PGN), the main component of the cell wall^4^. PGN is a heteropolymeric layer that completely encloses the bacterial cell and provides the bacterial shape and integrity. The biosynthesis of PGN requires several steps, which are evolutionarily conserved in all bacterial species but are missing in eukaryotic cells^5^. Therefore, it is an optimal target for antibacterial agents.

PGN synthesis occurs in three distinctive compartments of the bacterial cell, namely, the cytoplasm, the cytoplasmic membrane, and the cell surface^6^: (1) In the cytoplasm, lipid II synthesis takes place; lipid II is a PGN precursor composed of an undecaprenyl pyrophosphate (UPP) anchor, the two amino sugars *N*-acetylglucosamine (GlcNAc) and *N*-acetylmuramic acid (MurNAc) and a covalently attached pentapeptide^7^. (2) Lipid II is afterwards flipped to the extracellular space (or periplasm for gram-negative bacteria) and is still anchored to the membrane via UPP^8^. (3) Following this, the GlcNAc-MurNAc-pentapeptide subunit is incorporated into the nascent PGN, leaving UPP attached to the membrane. UPP is subsequently dephosphorylated to undecaprenyl phosphate (UP), which is flipped back into the cytoplasm and implemented into a new PGN synthesis cycle^9^.

This biosynthetic pathway has been shown to be an ideal target for antimicrobial compounds at any stage of (1)–(3)^4,10^. In many cases, the incorporation of lipid II into the nascent PGN layer is prevented; antibiotics either bind directly to lipid II or to enzymes that catalyze its incorporation into PGN. Both types of binding lead to nonrecycling of UP and subsequent inhibition of lipid II synthesis. As a consequence, bacterial cell growth is hindered.

**Figure 1:**
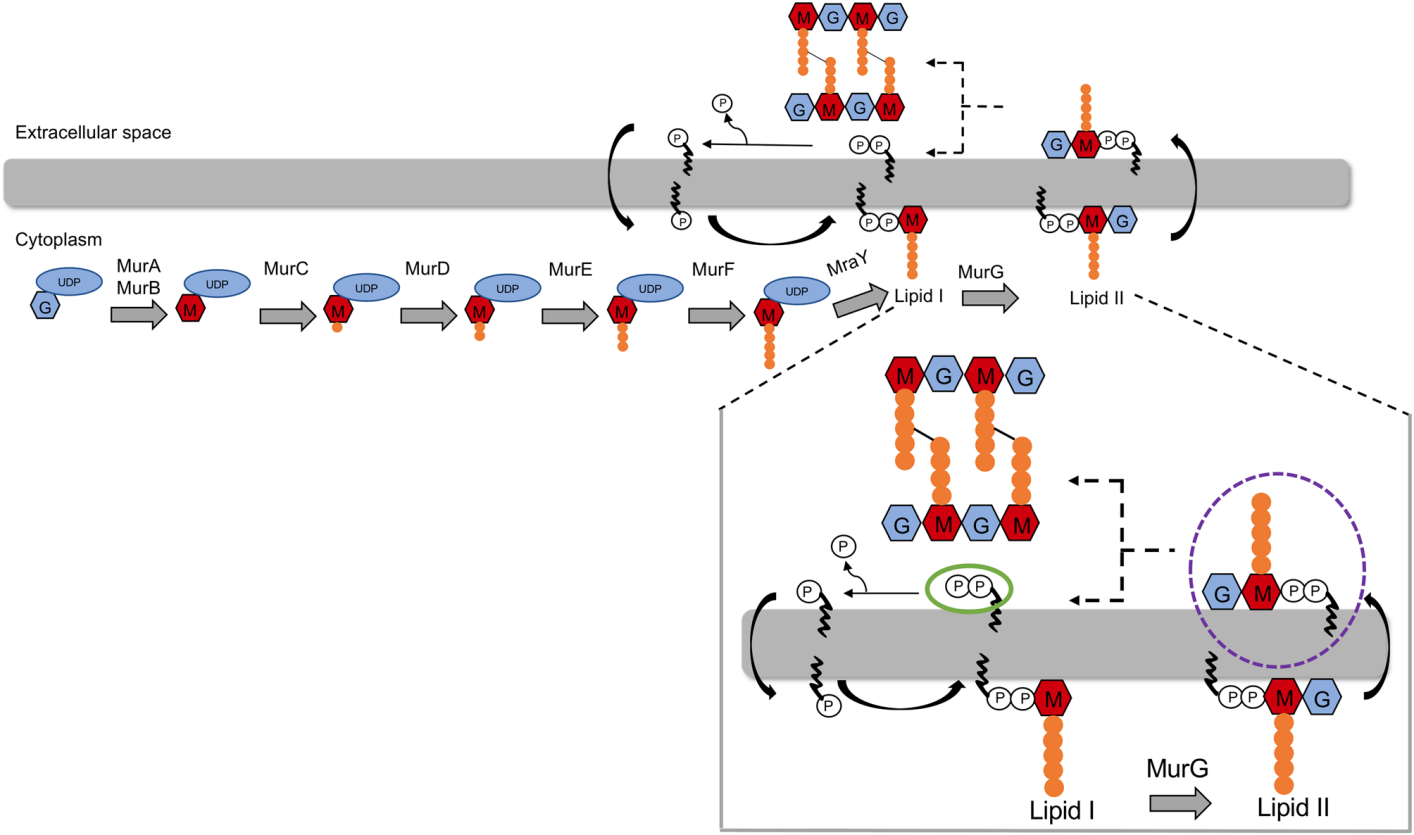
Schematic view of peptidoglycan synthesis. Synthesis of lipid II in the cytoplasm and its incorporation into the peptidoglycan. Phosphates are marked with a P, undecaprenyl as a black curved line, uridine phosphate (UDP) in light blue, GlcNAc in blue, MurNAc in red and amino acids of the pentapeptide in orange. Enlarged step of peptidoglycan synthesis showing targets of bacitracin, which is undecaprenyl pyrophosphate (green circle), and targets of many antimicrobial peptides, such as nisin, gallidermin, lysobactin, and ramoplanin: lipid II (violet, dotted circle). *GlcNAc N*-acetylglucosamine; *MurNAc N*-acetylmuramic acid. The figure was created using Microsoft Powerpoint Version 16.54.

Binding of antibiotics occurs to various moieties of lipid II, e.g., to the pyrophosphate moiety (lantibiotics such as nisin and gallidermin)^11–13^ or to the pentapeptide moiety (glycopeptides such as vancomycin)^10,14,15^ (Fig. 1). A particular class are small antimicrobial peptides (AMPs) that bind specifically to the pyrophosphate-sugar moiety of lipid II^11^, such as the lipoglycodepsipeptide ramoplanin and the acylcyclodepsipeptide lysobactin^10,16–18^.

In addition to lipid II binders, antibiotics are known to inhibit PGN biosynthesis at another stage, e.g., the cyclic peptide bacitracin. The binding of bacitracin to UPP inhibits the dephosphorylation of UP and blocks its regeneration, resulting in the accumulation of intracellular PGN precursors^19–21^ (Fig. 1). The net effect is the destabilization of the cell wall, leading to cell growth inhibition and subsequently to the death of the bacteria.

Bacitracin has been suggested to form a compact ternary 1:1:1 antibiotic-metal-lipid complex that, with its highly amphipathic structure, enhances membrane-binding affinity^20^. Due to a stabilizing effect and increased antimicrobial activity, a zinc-bacitracin (Zn-bacitracin) complex has been commonly used in human and veterinary medicine in antibiotic formulations^22,23^.

Bacitracin has also been used as a growth-promoting additive in animal feed^24^. Additionally, it has been shown to control necrotic enteritis effectively^25^ and is therefore used as a drug in many countries. Long-term usage of bacitracin in animals leads to an increase in resistance genes in microorganisms. Some molecular bacitracin resistance mechanisms have been reported in bacteria^26–29^.

One of the identified resistance mechanisms against AMP in human pathogens is based on the expression of bacitracin efflux (Bce) transporter, a member of the ABC transporter family. Bce confers high-level resistance to bacitracin and/or lantibiotics such as nisin and gallidermin in *Bacillus subtilis*, *Staphylococcus aureus* and *Streptococcus agalactiae*^27,30–34^. Genomic analysis revealed the presence of homologous transporters (BceAB-type transporters) mostly in bacteria predominantly found in soil and in human pathogenic bacteria^35^.

The first BceAB-type transporter was identified in *B. subtilis.* Adjacent to the *bceAB* genes, the *bceRS* genes are located, encoding a two-component system (TCS). The TCS regulates the expression of transporters^27^ and it is hypothesized that the detoxification against peptide antibiotics is functionally linked to it^36^. Status quo is that upon substrate binding the BceAB type transporter transfers a signal to the histidine kinase that then phosphorylates its cognate response regulator which induces the expression of the ABC transporter genes. This was described i.e. for the GraRS-VraFG system^37^ in *S. aureus* and also for several TCS-ABC transporters in *B. subtilis* (BceRS-AB, YxdJK-LM and YvcPQ-RS)^35,38^. The direct interaction between the BceAB transporter and the BceS histidine kinase was demonstrated in *B. subtilis*^36^. In that study, it was shown that BceAB, which was purified from the membrane, needs to form a complex with BceRS in order to initiate antibiotic resistance signalling^36^. A characteristic feature of these BceAB-type transporters is an extracellular domain (ECD) of roughly 210–230 amino acid located between transmembrane helices 7 and 8. These domains are supposed to be involved in binding the substrate (e.g., bacitracin). This is hypothesized due to the reason that the cognate histidine kinase, consists only of a short loop which is buried almost entirely in the cytoplasmic membrane and thus cannot detect extracellular stimuli^39^. So the binding to the ECD is supposed to trigger simultaneously the histidine kinase (HK)^36^. It has been shown with medically and biotechnologically relevant Gram-positive species that BceS-like HKs require BceAB-type transporters for antibiotic signaling^31,36,40,41^. Moreover, ATP hydrolysis by the ATPase BceA was shown to play an essential role for lantibiotic signaling^23^. Additionally, it was found that the associated sensor kinase BceS is unable to detect bacitracin in the absence of the transporter BceAB, which led to proposition that the transporter contains the involved sensory domain of the system^40,42^. More recently, the binding of AMP LL-37 to the ECD of VraG (a BceAB homologous transporter) was described in *B. subtilis*^37^.

Similar operon structures have been reported for other BceAB-type transporters, in some cases with an additional gene encoding a membrane-embedding protein such as *Sa*NSR, a BceAB-type transporter in the human pathogen *S. agalactiae*, conferring resistance against lantibiotics such as nisin A, nisin H and gallidermin^32,43^. Using a peptide release assay, it was postulated that *Sa*NsrFP transports these peptides via an efflux mechanism back into the medium^34^.

Several putative mechanisms for BceAB-type transporters have been proposed, ranging from AMP removal from the membrane^44^, functioning as an exporter^34^, to flipping the UPP^21^. Recently, a study proposed a target-AMP dissociative, ATP-hydrolysis-driven mechanism for BceAB-type transporters, in which the target-AMP complex is recognized and UPP physically released from the grip of bacitracin^45^.

In our study, in order to elucidate the mechanism of *Sa*NsrFP, we expressed the BceAB-type transporter *Sa*NsrFP in *L. lactis* NZ9000 without its cognate TCS^34^. We hypothesized that the transporter alone is able to sense an AMP in the surrounding since the Bce type transporters are known to play a crucial role in the signalling process^37,42,43,46^. It was shown for the related BceAB transporter that signalling is triggered by the activity of the transporter itself and the transporter can autoregulate its own production^46^. In previous work, it was shown that *Sa*NsrFP is able to confer resistance against nisin without its TCS^34^. As a control for our study, we also analyzed the ATPase-deficient mutant of the ABC transporter that showed no ATPase function in-vitro^47^.

Since the BceAB system of *B. subtilis* is known to confer resistance against bacitracin and also other antibiotics i.e. mersacidin, plectasine and actagardine^27,38^, we wanted to test whether the *Sa*NsrFP is also able to confer resistance against bacitracin and structurally different antibiotics. By investigating the effect the expression of *sansrfp* has on the proteome level, we gained further insights into the mode of action. Since here it was observed that genes involved in cell wall biosynthesis were downregulated, we also analyzed the composition of the peptidoglycan layer. Cell wall modification is one of several mechanisms of being involved in antibiotic resistance and has been shown to play an important role in the resistance mechanism in *S. aureus*, *C. difficile*, *S. pneumoniae* and *S. agalactiae*^48–50^. Also, in recent studies it is suggested that transporters can have a direct or indirect influence on peptidoglycan biosynthesis or peptidoglycan remodeling as was shown recently for the ABC transporter YtrBCDEF in *B. subtilis*^51^*.*

In this study, we aimed to characterize *Sa*NsrFP in larger detail and analyzed the ability of this transporter to confer resistance against different structurally unrelated compounds, such as lysobactin, ramoplanin, vancomycin and bacitracin, as well as its zinc complex Zn-bacitracin. Whole-cell proteome and cytosolic PGN precursor analysis supported our hypothesis that the different antibiotics bind to *Sa*NsrFP inducing an altering of the cell wall. This is relying on the ATP hydrolysis of *Sa*NsrFP since the ATP hydrolysis deficient mutant does not show this phenotype. The transporter is able to protect the target via a first-line and second-line defense, and the energy set free by ATP hydrolysis could be the key to resetting the system. Our study provides new insights into the resistance mechanism of the BceAB-type transporter *Sa*NsrFP. The data presented are in agreement with a mechanism of protection by shielding the target of the antimicrobial peptide.

## Results

### *Sa*NsrFP enables normal growth in the presence of bacitracin

The BceAB-type transporter NsrFP from the human pathogen *S. agalactiae* COH1, *Sa*NsrFP, has been shown to confer resistance against the lantibiotic nisin and structurally related compounds such as nisin H and gallidermin by recognizing and binding to the N-terminus of these lantibiotics^34^.

**Figure 2:**
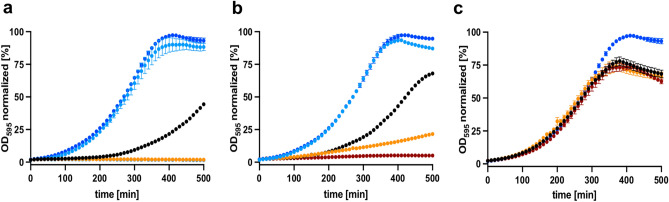
(**a**) Growth curve of the BceAB type ABC transporter expressing strain *L. lactis* NZ9000NsrFP (light blue), the ATP-hydrolysis deficient mutant strain *L. lactis* NZ9000NsrF_H202A_P (orange), the empty plasmid expressing strain *L. lactis* NZ9000Cm (black) and the nisin transporter expressing strain *L. lactis* NZ9000NisT (dark red) were induced with 0.3 nM nisin and treated with 1 µM bacitracin and 1 mM ZnCl_2_. As a control, *L. lactis* NZ9000NsrFP (dark blue) was induced with 0.3 nM nisin, and 1 mM ZnCl_2_ was added. (**b**) Growth curve of *L. lactis* NZ9000NsrFP (light blue), *L. lactis* NZ9000NsrF_H202A_P (orange), *L. lactis* NZ9000Cm (black), *L. lactis* NZ9000NisT (dark red) induced with 0.3 nM nisin and treated with an additional 4 µM bacitracin without ZnCl_2_. As a control, *L. lactis* NZ9000NsrFP (dark blue) was induced with 0.3 nM nisin. (**c**) Growth curve of the control, *L. lactis* NZ9000NsrFP (dark blue), *L. lactis* NZ9000Cm (black) and *L. lactis* NZ9000NisT (dark red) induced with 0.3 nM nisin, and 1 mM ZnCl_2_ was added. The normalized OD_600_ was plotted against the time using GraphPad Prism version 9.2.0 for Mac, GraphPad Software, San Diego, California USA, www.graphpad.com.

To investigate whether *Sa*NsrFP confers resistance against bacitracin and Zn-bacitracin, binding to the lipid carrier UPP^20,52^, we analyzed the influence of the expression of the *sansrfp* gene. The sensitive *L. lactis* strain NZ9000, served as an indicator strain for these studies which was transformed with a plasmid encoding the *nsrfp* gene. We included two controls where the strains were transformed with (I) an empty plasmid (*L. lactis* NZ9000Cm) (II) a plasmid containing a variant of the *nsrfp* gene (*L. lactis* NZ9000*Sa*NsrF_H202A_P)^34^. This NZ9000*Sa*NsrF_H202A_P strain is used since the transporter carries a mutation in the H-loop, a highly conserved region of ABC transporters, and as a result is not able to hydrolyze ATP^47^. This mutation causes loss of ATP hydrolysis and stabilization of the closed conformation^53^. Although the substrate still binds to it as the transporter, it cannot be translocated because the required energy cannot be provided^34,47^. The growth of *L. lactis* NZ9000Cm, *L. lactis* NZ9000*Sa*NsrFP, *L. lactis* NZ9000*Sa*NsrF_H202A_P and *L. lactis* NZ9000NisT was monitored online over a time period of 500 min (Fig. 2a,b). After adding the different antibiotics to the culture, the growth curve was determined.

The expression of the *sansrFP* and *sansrFP*_H202_A genes was induced by adding a sublethal concentration of 0.3 nM nisin to the cells. This subinhibitory concentration of nisin is able to induce the *nisA* promotor in the pIL-SV plasmids which enables the gene expression of the respective protein. Important to note that this low concentration of nisin is not harming the cells as observed by different growth studies^54,55^. Simultaneously, either 1 µM bacitracin in combination with 1 mM ZnCl_2_ (Fig. 2a) or 4 µM bacitracin without zinc (Fig. 2b) was added. As a control, all strains were only induced with 0.3 nM nisin without receiving any additional supplements (Fig. 2c).

Severe growth inhibition was shown for the *L. lactis* NZ9000Cm *and L. lactis* NZ9000NisT strains. When using the *L. lactis* NZ9000*Sa*NsrFP strain, however, the growth behavior was comparable to that of *L. lactis* NZ9000Cm without the addition of bacitracin (Fig. 2a,b light blue curve and c). Interestingly, *L. lactis* NZ9000*Sa*NsrF_H202A_P cells were unable to grow when treated with (Zn)-bacitracin (Fig. 2a orange), whereas reduced growth was observed when bacitracin was added (Fig. 2b orange). Growth retardation, as observed in *L. lactis* NZ9000Cm upon the addition of bacitracin, has been shown for many bacterial cells, such as methicillin-resistant *S. aureus* and group B streptococci^56,57^. It is caused by the binding of bacitracin to UPP, preventing the dephosphorylation reaction and leading to the interruption of PGN biosynthesis^20^. Intriguingly, *L. lactis* NZ9000*Sa*NsrFP did not show reduced growth (Fig. 2a,b light blue) in the presence of bacitracin in comparison to the control strain *L. lactis* NZ9000*Sa*NsrFP (Fig. 2a,b blue) and the sensitive strain *L. lactis* NZ9000Cm.

These results demonstrated that *S*aNsrFP is involved in bacitracin resistance with a requirement for ATP hydrolysis. For our study, we expressed *sansrfp* without its TCS, leading us to the conclusion that the transporter alone is directly involved in bacitracin resistance.

### *Sa*NsrFP confers resistance against bacitracin, ramoplanin, vancomycin and lysobactin

Since *Sa*NsrFP conferred resistance in addition to lanthipeptides against bacitracin and Zn-bacitracin, we extended the resistance study to a structurally diverse, rather unrelated group of antibiotics, including ramoplanin, vancomycin and lysobactin, all of which bind to different parts of lipid II^10,18^.

After adding the different antibiotics to the cultures of the strains *L. lactis* wild type (WT), *L. lactis* NZ9000Cm, *L. lactis* NZ9000*Sa*NsrFP and *L. lactis Sa*NsrF_H202A_P, the IC_50_ was determined. By dividing the IC_50_ obtained for the strains producing *Sa*NsrFP or the inactive variant SaNsrF_H202A_P by the IC_50_ value obtained for the sensitive strain NZ9000Cm, the fold change of resistance was calculated, which was independent of small variations in bacterial cell growth behavior.

**Figure 3:**
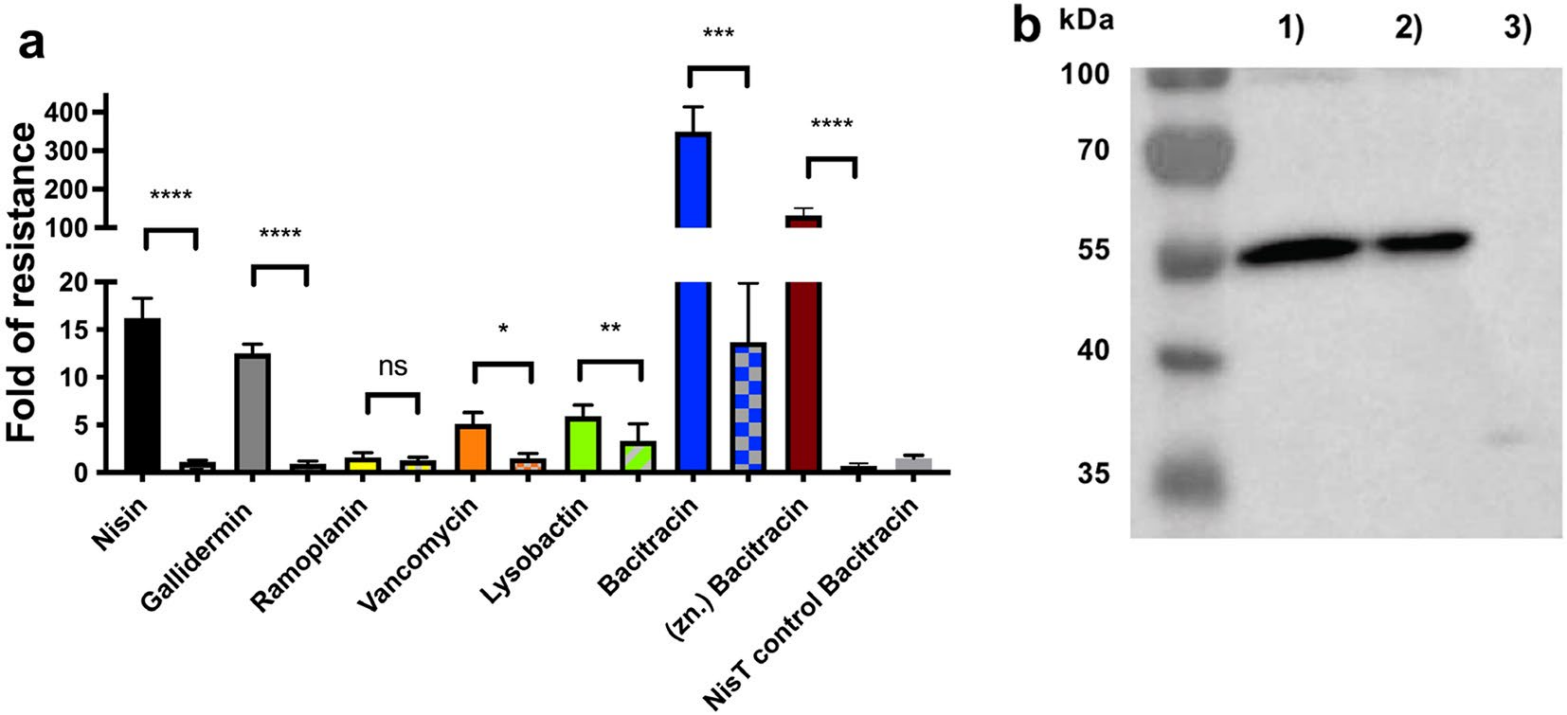
(**a**) Fold of resistance of *L. lactis *NZ9000NsrFP and NZ9000NsrF_H202A_P (hatched bars) against *L. lactis *NZ9000Cm calculated with the determined IC_50_ of ramoplanin A2 (yellow), vancomycin (orange), lysobactin (green), bacitracin (blue) and bacitracin with ZnCl_2_ (dark red). Values for nisin and gallidermin were taken from Reiners et al.^34^ and marked with an asterisk. Values were calculated from at least 4 independent measurements and are also listed in Table 1. A two-sided Students t-test was performed with the IC_50_ data obtained for *Sa*NsrFP and *Sa*NsrF_H202A_P. Significance was marked with an asterisk. p-values were listed in a separate table (SI Table S1) in the supplement. (**b**) Expression of *Sa*NsrFP (1) and *Sa*NsrF_H202A_P (2) and the empty vector pIL-SV (3) in *L. lactis* NZ9000, monitored *via* western blot with a polyclonal antibody against the extracellular domain of *Sa*NsrP. Loaded are purified membranes from the corresponding strains. A nonlinear regression curve fit and a two-sided, unpaired Students t-test was performed using Graphpad Prism version 9.2.0 for Mac, GraphPad Software, San Diego, California USA, www.graphpad.com.

Compared to *L. lactis* WT, *L. lactis* NZ9000SaNsrFP exhibited small resistance to the lipid II binders, vancomycin and lysobactin (two to sixfold) (Fig. 3a, SI Fig. S1, Table 1). No significant differences were detected between NZ9000NsrFP and NZ9000NsrFH202AP with ramoplanin. The fold increases of resistance (two, fivefold increases) obtained were significantly lower than the fold changes described for nisin (16-fold) and gallidermin (12-fold)^34^, suggesting that nisin is a preferred substrate of the transporter. IC_50_ values decreased to similar levels in *L. lactis* NZ9000SaNsrF_H202A_P (one, two and threefold), as observed for the *L. lactis* NZ9000Cm strain (73.0 nM, 213.5 nM, and 30.7 nM, respectively, Table 1), indicating that resistance was not achieved solely by the expression of the SaNsrF_H202A_P transporter. To ensure that this loss of resistance was not due to different production levels, we performed Western blot analysis on the purified membrane fractions of the transporter expressing cells using a polyclonal antibody against the ECD of *Sa*NsrP (Fig. 3b). We confirmed that comparable levels of transporters were produced in both strains. Since ATP hydrolysis activity was deleted in the SaNsrF_H202A_P mutant, the obtained results suggested that *Sa*NsrFP requires ATP hydrolysis to confer resistance.

**Table 1:** Measured IC_50_ values and calculated fold of resistance for the antibiotics ramoplanin A2, vancomycin, lysobactin and bacitracin and for the strains NZ9000Cm, NZ9000*Sa*NsrF_H202A_P and NZ9000*Sa*NsrFP.

Antibiotic	*L. lactis* NZ9000Cm	*L. lactis* NZ9000NsrFP		*L. lactis* NZ9000NsrF_H202A_P		*L. lactis* NZ9000NisT	
	IC_50_ (nM)	IC_50_ (nM)	Fold of resistance	IC_50_ (nM)	Fold of resistance	IC_50_ (nM)	Fold of resistance
Ramoplanin A2	73 ± 18	121 ± 34	2 ± 1	92 ± 21	1 ± 0	95	1.3 ± 0
Vancomycin	214 ± 27	1078 ± 264	5 ± 1	325 ± 117	2 ± 1	133 ± 9	1 ± 0
Lysobactin	31 ± 17	182 ± 37	6 ± 1	101.8 ± 52.2	3 ± 2	25 ± 3	1 ± 0
Bacitracin	938 ± 94	327,500 ± 60,884	349 ± 65	12,855 ± 8517	14 ± 9	733	1 ± 0
Bacitracin ZnCl_2_	81 ± 16	10,694 ± 1541	132± 19	58.7 ± 17.9	1 ± 0	49 ± 16	1 ± 0

The control strain NZ9000NisT was only treated with bacitracin or (Zn)-bacitracin. Each measurement was performed at least by 3 biological replicated with 3 technical replicates each.

However, high-level resistance was observed for *L. lactis* NZ9000SaNsrFP to bacitracin (350-fold) and (Zn)-bacitracin (132-fold) compared to *L. lactis* WT (Fig. 3a, SI Fig. S1, Table 1). In contrast, *L. lactis* NZ9000*Sa*NsrF_H202A_P displayed only moderate resistance (13-fold), which was completely abolished when the cells were treated with (Zn)-bacitracin (0.7-fold). Resistance at a low level against nisin (2.6-fold) was shown in Khosa et al., in which an inactive variant of the protease *Sa*NSR (*Sa*NSR_S236A_) was produced^32^. Even though, it was demonstrated that NZ9000*Sa*NsrF_H202A_P shows no ATPase activity in-vitro^47^, it is known that it is difficult to compare in-vitro with in-vivo data since it cannot be excluded that other processes in the bacterial cell might lead to such a residual bacitracin resistance for *Sa*NsrF_H202A_P. On the other hand, the residual resistance is only observed with bacitracin not with zinc-bacitracin. It has been shown that bacitracin shows a higher attraction to the membranes in the presence of zinc and the target most probably due to the observation that it is forced into an amphiphile conformation^20^. This could explain why only residual resistance is observed with only bacitracin since it cannot access the membrane as easily as with zinc.

To strengthen this hypothesis and to exclude that the resistance to bacitracin in *L. lactis* NZ9000SaNsrF_H202A_P is caused by an altered membrane protein composition due to the overexpression, we performed growth inhibition experiments with another large ABC transporter, namely, NisT from *L. lactis,* which is not present in the genome of the NZ9000 strain used, using the same plasmid backbone*.* Recently, it was shown that NisT is produced in high amounts in the used strain^58,59^. However, NisT is not relevant to bacitracin resistance, as evidenced by similar IC_50_ values of the strain NZ9000NisT, producing no NisT (Table 1). Therefore, it can be concluded that the production of large membrane proteins, as well as ATP hydrolysis and a possible alteration of the membrane protein composition is not the explanation for the resistant phenotype but is due to the production of *Sa*NsrFP or its inactive variant.

### *Sa*NsrFP prevents the accumulation of peptidoglycan precursors after the addition of bacitracin

**Figure 4:**
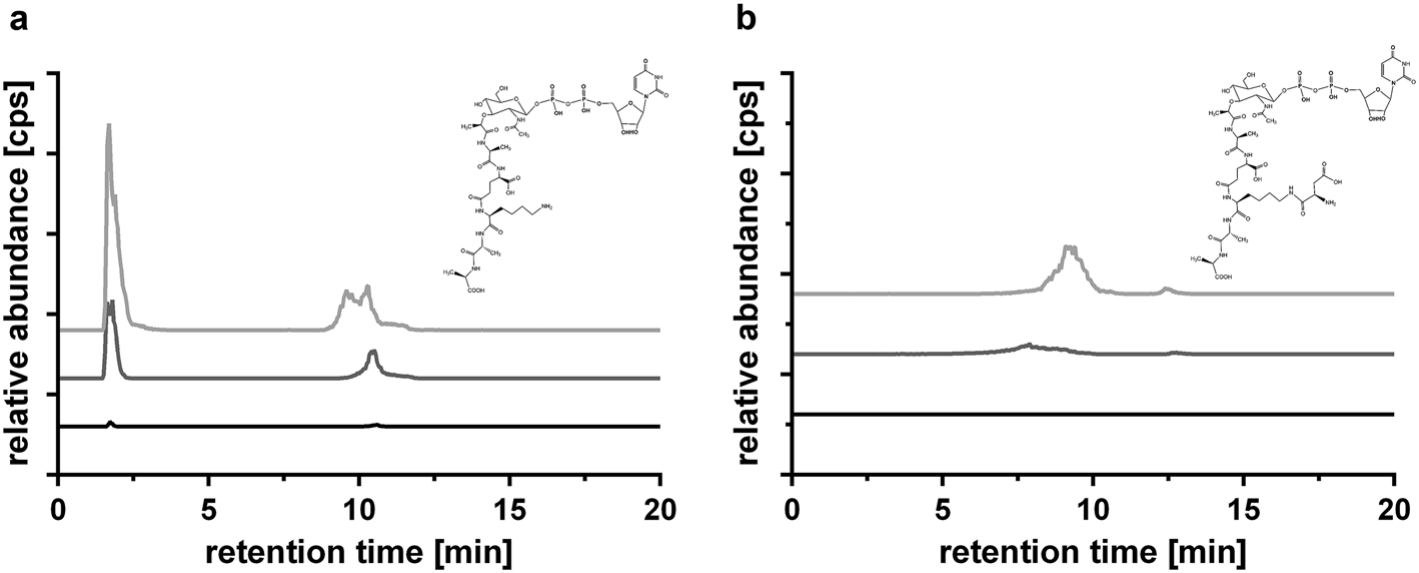
Relative abundance of obtained mass in cps against retention time in min of precursor accumulation after treatment with bacitracin. (**a**) Extracted ion chromatography (EIC) spectrum for UDP-MurNAc-l-Ala-d-iGlu-l-Lys-d-Ala-d-Ala (1148.4 m/z^−1^) and (**b**) EIC spectrum for UDP-MurNAc-l-Ala-d-iGlu-l-Lys(d-Asp)-d-Ala-d-Ala (1263.4 m/z^−1^) of the strains NZ9000Cm (light gray), NZ9000*Sa*NsrF_H202A_P (gray) and NZ9000*Sa*NsrFP (black). Extracted ion chromatograms (EICs) in negative ion mode for UDP-MurNAc-l-Ala-d-iGlu-l-Lys-d-Ala-d-Ala (m/z^−1^ 1148.34 ± 0.1) and UDP-MurNAc-l-Ala-d-iGlu-l-Lys-(d-Asp)-d-Ala-d-Ala (m/z^−1^ 1263.37 ± 0.1) were analyzed with Data Analysis (Bruker), exported and presented with GraphPad Prism 6.0, GraphPad Software, San Diego, California USA, www.graphpad.com. *UDP* undecaprenyl-phosphate, *MurNAc N*-acetylmuramic acid, *Ala* alanine, *iGlu* isoglutamic acid, *Glu* glutamine, *Asp* aspartate/aspartic acid, *Lys* lysine.

To further understand the mechanism of action of *Sa*NsrFP, we analyzed PGN precursor accumulation in the cytoplasm of *L. lactis* NZ9000*Sa*NsrFP, *L. lactis* NZ9000Cm and *L. lactis* NZ9000*Sa*NsrF_H202A_P grown in the presence of bacitracin. HPLC/MS analysis of the PGN extracts revealed the presence of the characteristic PGN precursors UDP-MurNAc-l-Ala-d-iGlu-l-Lys-d-Ala-d-Ala (1148.4 m/z^−1^) and MurNAc-l-Ala-d-iGlu-l-Lys-(d-Asp)-d-Ala-d-Ala (1263.4 m/z^−1^) in the *L. lactis* NZ9000Cm strain (Fig. 4a,b light gray).

Interestingly, no accumulation of the PGN precursors was observed in the *L. lactis* NZ9000*Sa*NsrFP strain (Fig. 4a,b black line), whereas *L. lactis* NZ9000*Sa*NsrFP without bacitracin treatment revealed the accumulation of the PGN precursor UDP-MurNAc-l-Ala-d-iGlu-l-Lys-d-Ala-d-Ala (1148.4 m/z^−1^) (SI Fig. S6a,b). The fact that bacitracin was not able to block UPP recycling, together with the results obtained in the resistance test, clearly suggests that *Sa*NsrFP prevents the binding of bacitracin to UPP and thus the accumulation of PGN precursors. This hypothesis is further supported by the results obtained for the *L. lactis* NZ9000*Sa*NsrF_H202A_P strain. Here, reduced PGN precursor accumulation was observed compared to the bacitracin-sensitive *L. lactis* NZ9000Cm strain (Fig. 4a,b gray line), implicating that the availability of UPP for bacitracin binding is decreased in the *L. lactis* NZ9000*Sa*NsrF_H202A_P strain. Considering that bacitracin did not inhibit PGN synthesis in *L. lactis* NZ9000*Sa*NsrFP, *Sa*NsrFP might protect the bacitracin target and directly interact with a component of PGN, most likely UPP, to evade the accumulation of PGN precursors.

Binding of bacitracin to the UPP or UP normally results in the accumulation of lipid II precursors in the cytosol. The lack of this accumulation in *L. lactis Sa*NsrFP suggests that bacitracin is unable to bind to its membrane-localized target UPP. This, together with the observation that the cell growth of *L. lactis Sa*NsrFP in the presence of bacitracin is similar to that of cells without bacitracin, supports the shielding mechanism proposed by Kobras et al.^45^.

### *Sa*NsrFP causes downregulation of proteins involved in peptidoglycan synthesis

**Figure 5:**
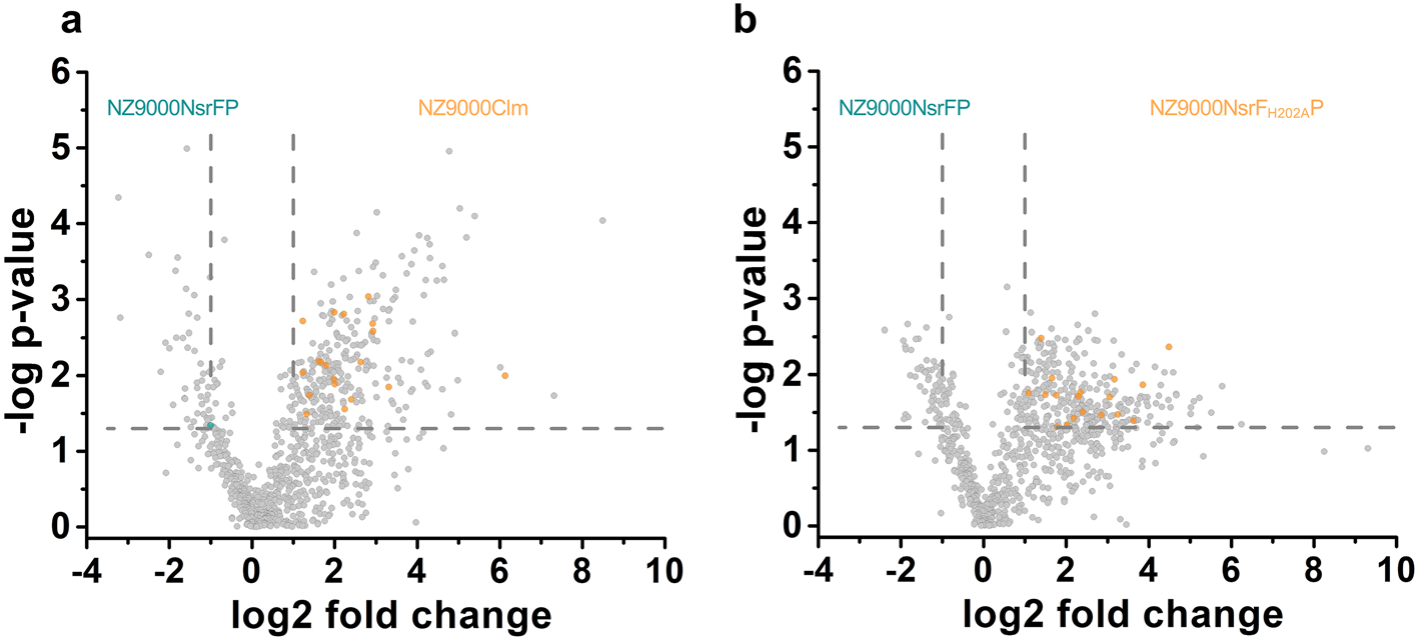
(**a**) Volcano plot of the proteome analysis of NZ9000*Sa*NsrFP against NZ9000Cm and (**b**) NZ9000*Sa*NsrFP against NZ9000*S*aNsrF_H202A_P. Proteins involved in cell wall synthesis are highlighted in orange if upregulated in NZ9000Cm (**a**) and NZ9000*Sa*NsrF_H202A_P (**b**) and highlighted in blue if upregulated in NZ9000*Sa*NsrFP. Proteins with a p-value ≤ 0.05 and a fold change ≥ 2 were considered as statistically significant. Proteome data was plotted using GraphPad Prism 6.0 for Mac, GraphPad Software, San Diego, California USA, www.graphpad.com.

To get an insight to the mode of action initiated by *Sa*NsrFP expression, we analyzed the whole proteome of *L. lactis* NZ9000*Sa*NsrFP, *L. lactis* NZ9000Cm and *L. lactis* NZ9000*Sa*NsrF_H202A_P grown under identical growth conditions (at 30 °C in GM17 medium containing 5 µg/ml chloramphenicol and induced with 0.3 nM nisin) by mass spectrometry. The analyses led to the identification of 894 proteins (identified by at least two unique peptides in each strain). The comparison between *L. lactis* NZ9000Cm and NZ9000*Sa*NsrFP revealed 315 with differential abundances (Fig. 5a) and 339 proteins showing differential abundances between the *L. lactis* strains NZ9000*Sa*NsrF_H202A_P and NZ9000*Sa*NsrFP (Fig. 5b). In 231 proteins there was no change of abundance. Here, we took the slightly different OD after 5 h of cell growth into account and adjusted the whole cell protein concentration accordingly. In particular, the latter highlights that these up- or downregulation of the proteins do not arise from the expression of the transporter since they are expressed at similar levels (see Fig. 2b). This high number of differentially produced proteins implied that the *L. lactis* NZ9000*Sa*NsrFP strain has to respond significantly to counteract the effects mediated by the presence and activity of the *Sa*NsrFP BceAB-type ABC transporter.

In-depth analysis showed that the production of proteins involved in PGN synthesis was reduced in *L. lactis* NZ9000*Sa*NsrFP (Fig. 4a,b, SI Fig. S3). Among them the UDP-N-acetylglucosamine 1-carboxyvinyltransferase MurA (ADJ59532), was produced 6.2-fold less in the *L. lactis* NZ9000*Sa*NsrFP compared to *L. lactis* NZ9000Cm; UDP-*N*-acetylmuramate-l-alanine ligase MurC (ADJ61283), 4.0-fold, UDP-*N*-acetylmuramoyl-l-alanyl-d-glutamate synthetase MurD (ADJ59924) the 3.5-fold, UDP-*N*-acetylmuramoylalanyl-d-glutamate-2,6-diaminopimelate ligase MurE (ADJ60966), 4.0-fold less produced in the control strain as well as UDP-*N*-acetylmuramoylalanyl-d-glutamyl-2,6-diaminopimelate-d-alanyl-d-alanine ligase MurF (ADJ59966). Furthermore, proteins involved in the synthesis of components of lipid II synthesis, such as uracil phosphoribosyl transferase or glucosamine-fructose-6-phosphate aminotransferase, responsible for UMP and glucosamine-6-phosphate synthesis, respectively, were downregulated in *L. lactis* NZ9000*Sa*NsrFP (Table 2, SI Fig. S3).

**Table 2:** Selected proteins of the proteome analysis with their description and their fold of down regulation in NZ9000*Sa*NsrFP compared to NZ9000Cm and NZ9000*Sa*NsrF_H202A_P.

Protein	Description	Fold of expression of *Sa*NsrFP in comparison to			
		NZ9000Cm	p-value	NZ9000*Sa*NsrF_H202A_P	p-value
ADJ59532	UDP-*N*-acetylglucosamine 1-carboxyvinyltransferase MurA	1.2		2.1	0.018
ADJ61283	UDP-*N*-acetylmuramate-l-alanine ligase MurC	4.0	0.013		
ADJ59924	UDP-*N*-acetylmuramoyl-l-alanyl-d-glutamate synthetase MurD	3.5	0.007	9.0	0.012
ADJ60966	UDP-*N*-acetylmuramoylalanyl-d-glutamate-2, 6-diaminopimelate ligase MurE	4.0	0.001	4.9	0.019
ADJ59382	UDP-*N*-acetylmuramoylalanyl-d-glutamyl-2,6-diaminopimelate-d-alanyl-d-alanine ligase MurF	2.5	0.032	4.5	0.037
ADJ60503	Glucosamine-fructose-6-phosphate aminotransferase	7.6	0.002	12.4	0.041
ADJ61146	Uracil phosphoribosyltransferase	2.4	0.001	2.1	
ADJ59249	UDP-galactopyranose mutase	3.9	0.011	7.2	0.034
ADJ59465	a-d-glucosamine 1,6-phosphomutase	1.0		1.0	
ADJ61162	Phenylalanyl-tRNA synthetase subunit beta	1.0		1.0	

The number of replicates were n = 5. We performed an ANOVA test and the p values are also part of the protein lists.

Only significant p-values were listed.

We included in the analysis proteins from other metabolic pathways, such as amino sugar metabolism and translation, represented by α-d-glucosamine-1,6-phosphomutase or phenylalanyl-tRNA synthetase beta subunit in order to show that the expression of the ABC transporter *Sa*NsrFP is not toxic to the cells and has an influence on other important cell processes. These proteins did not reveal any differences in production among all three tested strains (Table 2), confirming that the observed differences for the other proteins were due to the expression of the active *Sa*NsrFP transporter.

**Figure 6:**
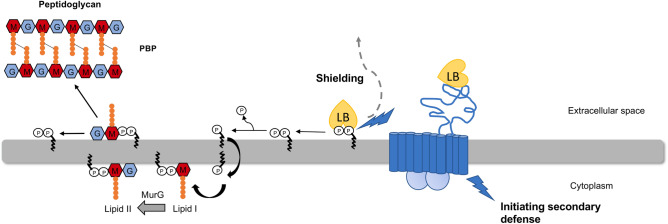
Schematic view of the proposed mechanism of *Sa*NsrFP. Phosphates are marked with a P, undecaprenyl as a black curved line, GlcNAc in blue, MurNAc in red and amino acids of the pentapeptide in orange. The transporter *Sa*NsrFP is shown in blue, showing its functions of sensing antibacterial attack, shielding the target most likely by releasing the target from the grip of bacitracin and initiating a secondary defense leading to possible cell wall thickening, modifying the electrostatic charge of the cell wall by integrating lipoteichoic acids and increasing d-alanylation in the cell wall. Subsequentially, the released target can enter a new cell wall synthesis cycle and be incorporated into the peptidoglycan (not shown fully here but in Fig. 1). *GlcNAc N*-acetylglucosamine, *MurNAc N*-acetylmuramic acid. The figure was created using Microsoft Powerpoint Version 16.54.

In summary, upregulated proteins when *Sa*NsrFP was produced, such as UDP-glucose-4-epimerase, *N*-acetylmuramic acid-6-phosphate etherase MurQ and RodA, were found to be associated with AMP resistance and cell wall modification (Fig. 6).

**Figure 7:**
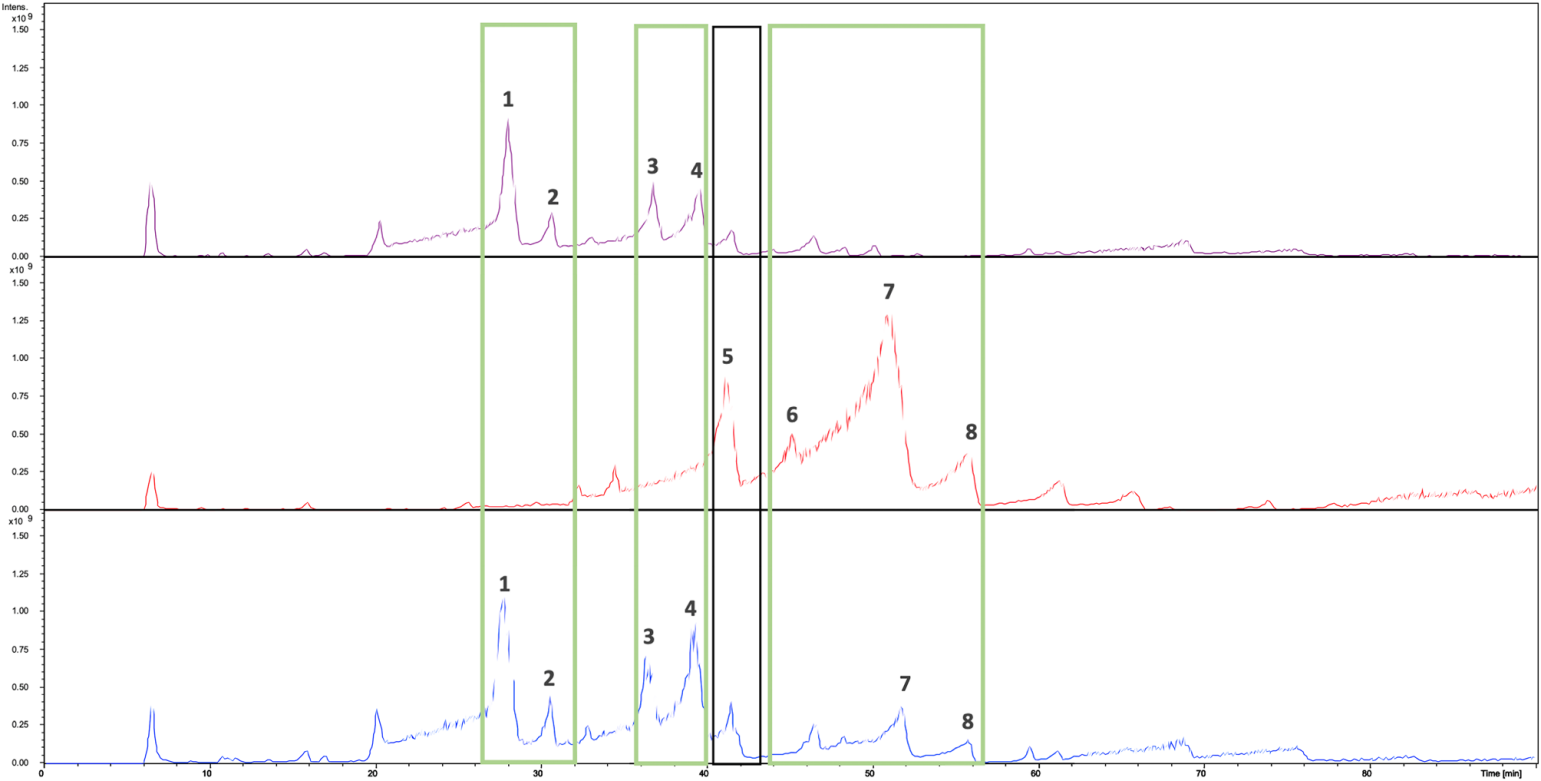
LC–MS chromatogram of isolated muropeptides from *L. lactis* NZ9000Cm (purple), *L. lactis* NZ9000NsrFP (red) and *L. lactis* NZ9000NsrF_H202A_P (blue). The peaks that only occur in *L. lactis* NZ9000Cm and *L. lactis* NZ9000NsrF_H202A_P are framed in green. Black framed is the peak, which can especially be observed in *L. lactis* NZ9000NsrFP. Peak 1: GlcNAc-MurNAc-l-Ala-d-iGln-l-Lys-(d-Asn); peak 2: GlcNAc-MurNAc-l-Ala-d-iGln-l-Lys-(d-Asp); peak 3: GlcNAc-MurNAc-l-Ala-d-iGln-l-Lys-(d-Asn)-d-Ala; peak 4: GlcNAc-MurNAc-l-Ala-d-iGln-l-Lys-(d-Asn)-d-Ala-d-Ala; peak 5: GlcNAc-MurNAc-l-Ala-d-iGln-l-Lys-(Ala)-d-Ala-d-Ala, and peaks 6–8: GlcNAc-MurNAc-l-Ala-d-iGln-l-Lys-(Ala-Ala)-d-Ala-d-Ala. *UDP* undecaprenyl-phosphate, *GlcNAc N*-acetylglucosamine, *MurNAc N*-acetylmuramic acid, *MurN N*-deacetylated muramic acid, *Ala* alanine, *iGlu* isoglutamic acid, *iGln* isoglutamine, *Glu* glutamine, *Asp* aspartate/aspartic acid, *Asn* asparagine, *Lys* lysine.

Additionally, the components of the nascent cell wall of *L. lactis* NZ9000Cm-, *Sa*NsrFP- and *Sa*NsrF_H202A_P-expressing *L. lactis* cells were analyzed via LC–MS. The comparison of their chromatograms revealed that some peaks, which are occurring only in the sensitive strain and the inactive mutant (Fig. 7, SI Fig. S5a–f). These peaks correspond to components consisting of GlcNAc-MurNAc-l-Ala-d-iGln-l-Lys-(d-Asn) with *m/z* 938.37 [M + H]^+^ (RT 27.0–28.3 min) (Fig. 7, SI Fig. S5a, peak 1), to GlcNAc-MurNAc-l-Ala-d-iGln-l-Lys-(d-Asp) with *m/z* 939.37 [M + H]^+^ (RT 29.8–30.6 and 32.4–33.1 min) (Fig. 7, SI Fig. S5b, peak 2), to GlcNAc-MurNAc-l-Ala-d-iGln-l-Lys-(d-Asn)-d-Ala with *m/z* 1009.45 [M + H]^+^ (RT 35.8–36.9 min) (Fig. 7, SI Fig. S5c, peak 3), to GlcNAc-MurNAc-l-Ala-d-iGln-l-Lys-(d-Asn)-d-Ala-d-Ala with *m/z* 1080.50 [M + H]^+^ (RT 38.0–39.3 min) (Fig. 7, SI Fig. S5d, peak 4). The obtained masses are in agreement with the data described in the literature^60,61^.

In contrast, one peak at RT 40.4–41.4 min with mass *m/z* 1037.49 [M + H]^+^ (Fig. 7, SI Fig. S5e, peak 5) was detected only in *L. lactis Sa*NsrFP-expressing cells. The detected mass may indicate the presence of GlcNAc-MurNAc-l-Ala-d-iGln-l-Lys-(Ala)-d-Ala-d-Ala muropeptide fragment, that could contain an Ala residue attached to Lys, forming the crosslinking bridge. In addition, we detected three peaks (Fig. 7, SI Fig. S5f, peaks 6–8) whose masses presumably corresponded to GlcNAc-MurNAc-l-Ala-d-iGln-l-Lys-(Ala-Ala)-d-Ala-d-Ala (RT 44.4–45.5 min, 47.0–51.8 min and 54.4–55.7 min) with *m/z* 1108.53 [M + H]^+^.These masses are in accordance with the masses described in the literature^62–64^. Intriguingly, the *Sa*NsrF_H202A_P-expressing strain also showed some double alanine muropeptide species, albeit with a lower percentage than in the active transporter. These findings suggest that the transporter not only confers resistance by defending the target but also induces modulation of the cell wall.

## Discussion

To elucidate the mechanism of the *Sa*NsrFP resistance mechanism, we showed that this transporter is able to circumvent reduced cell growth when cells are treated with bacitracin and/or Zn-bacitracin. Furthermore, we determined that resistance occurs against lipid II-binding AMPs. However, the highest resistance was observed for bacitracin and its Zn-bacitracin counterpart. This suggesting that this is the main substrate for NsrFP. The previously observed nisin resistance (Reiners et al.^34^) appears to be a side effect of the resistance mechanism. Here, our data implies that this resistance is ATP hydrolysis dependent and therefore is an active process, something that has been underestimated until now. Bacitracin resistance has been shown for several other BceAB-type transporters and appears to be conserved within this protein family. Examples include the AnrAB transporter from *Listeria monocytogenes*^30^, VraDE from *S. aureus*^31,33^, and the ABC transporter BceAB from *B. subtilis*^38^. However, similar to *Sa*NsrFP, these transporters additionally exhibited a certain degree of resistance to nisin and gallidermin, suggesting a general resistance mechanism rather than specific resistance to one type or even to specific antibiotics. Therefore, considering that *Sa*NsrFP confers resistance to structurally unrelated compounds, we concluded that *Sa*NsrFP is neither able to inactivate nor bind various compounds but rather that the resistance is based on a more general mechanism, such as shielding PGN biosynthesis components, including lipid II, UPP or UP, which are exposed on the outer surface of the bacterial membrane. The high-level resistance observed for the UPP binders bacitracin and (Zn)-bacitracin suggested that *Sa*NsrFP could shield either UPP or lipid II. (Figs. 1, 3a). Current hypotheses explain the resistance mechanism by the inaccessibility of the target UPP to bacitracin in this strain by either (i) target removal^21^, (ii) target protection^45^ or (iii) the combination of an active AMP defense mechanism that also mediates a multifactorial AMP defense response. The AMP defense mechanism does not involve only a higher expression of ABC transporter encoding genes but also modifications of the cell wall structure or the membrane lipid composition, PGN thickening, changes in net charge and degrading enzymes^4^. Given that *L. lactis Sa*NsrFP cells are still growing at high (Zn-) bacitracin concentrations, PGN synthesis was not completely inhibited. Since *Sa*NsrFP cells repel structurally diverse antibiotics, as is known for the related BceAB transporter of *B. subtilis*^27^*,* we hypothesize that *Sa*NsrFP could mediate resistance by shielding UPP and subsequent modification of PGN synthesis. These mechanisms have also been postulated by recent studies^45^. Based on previous studies and transporter activity studies in the presence of accumulated UPP or C35 isoprenoid heptaprenyl diphosphate (HPP), the authors proposed that the BceAB transporter detects UPP-bacitracin complexes and shields the target (e.g., lipid II or UPP or HPP) by severing the bond between them^65^. This further excludes other mechanisms, such as UPP flipping for BceAB in *B. subtilis*^21^ and import and inactivation of the target^23^.

Current opinion of researchers investigating antibiotic resistance conferring ABC transporters is that detoxification against peptide antibiotics is functionally linked to a two-component system^36^. It is hypothesized that upon sensing the antibiotic, the histidine kinase phosphorylates its cognate response regulator which induces the expression of the ABC transporter genes. Such a scenario was described i.e., for the GraRS-VraFG system^37^ in *S. aureus* and also for several TCS-ABC transporters in *B. subtilis* (BceRS-AB, YxdJK-LM and YvcPQ-RS)^36,38^. Moreover, a direct interaction of the BceRS and BceAB was shown in in vitro and in vivo studies^36^. In their study, it is claimed that BceAB and the TCS need to form a complex in order to be able to sense the AMP.

In our study, in order to elucidate the mechanism of the BceAB-type transporter *Sa*NsrFP, we expressed it without its cognate TCS. It has been shown that the ABC transporter without its TCS can confer resistance against nisin^34^. The large extracellular domain is the hallmark of BceAB-type transporter which is hypothesized to be involved in extracellular detection of antibiotics^43^. Interestingly, the cognate histidine kinase consists only of a short loop which is buried almost entirely in the cytoplasmic membrane and thus cannot detect extracellular stimuli^39^. The crucial role of a Bce-type transporter for lantibiotic signalling has been shown in various studies already^23,42^. For the BceAB transporter it was shown that signalling is triggered by the activity of the transporter itself and the transporter can autoregulate its own production^45,46^. This is the reason we hypothesize that the ABC transporter *Sa*NsrFP should also be able to sense the AMP via its large extracellular domain. Therefore, we strived to investigate the role *Sa*NsrFP plays together with its 221 amino acid large extracellular domain in conferring resistance without its cognate TCS.

Since *Sa*NsrFP is able to confer resistance against bacitracin and other AMP’s, we can show that the transporter is directly involved in sensing the antibiotic and the resistance process.

By the expression of *sansrfp*, adjustments within the bacterial cells occur. For example, the downregulation of proteins involved in lipid II biosynthesis. The reduced production of the key enzymes of the lipid II cycle was remarkable and suggested that the biosynthesis of new lipid II molecules occurred with less efficiency in the *L. lactis* NZ9000*Sa*NsrFP strain. This could be the case if lipid II or UPP might be the actual substrate of *Sa*NsrFP, but this hypothesis remains controversial, as it does not correspond to the growth behavior observed in the growth analysis. Here, the *Sa*NsrFP-expressing strain showed similar growth to the control strains *L. lactis* NZ9000NisT and *L. lactis* NZ9000Cm (see above Fig. 2c).

This strengthens the idea that BceAB-type transporters interact with precursors of cell wall synthesis or its recycling by binding. Therefore, the lipid II cycle might be inhibited, and the bacteria react by downregulating its lipid II pool. This has been directly shown by the analyses of the cell wall precursors that were clearly reduced in the *Sa*NsrFP strain. This would not only result in growth inhibition but would also lower the number of available targets at the membrane surface. Controversially, we could not see reduced growth in *Sa*NsrFP-expressing cells, and thus far, it was not possible to measure a difference in targets at the membrane surface. Less target on the surface could explain the moderate resistance observed for the lipid II binding AMPs like ramoplanin, vancomycin and others but it is not at all clear if this is a possible scenario since the removal of the target would likely lead to growth inhibition which would be toxic for the cell. Therefore, we cannot entirely exclude that the heterologous expression of the BceAB-type transporter influences the cell wall synthesis of *L. lactis* or whether the transporter itself is responsible for the alteration of its cell wall. Nonetheless, the NICE expression system (nisin-controlled gene expression system) that we used for the overexpression of *Sa*NsrFP is a tightly regulated system which can be turned on by adding a subinhibitory concentration of nisin to the media. It has been shown for the system that genes of closely related Gram-positive organisms (e.g., Streptococcus, Enterococcus, Staphylococcus, and low-GC Lactobacillus) are expressed effectively usually without any problems^66^. Interestingly, in the work of Marreddy et al.^67^, overexpression of a membrane protein led to an upregulation of cell wall synthesis in the membrane protein expressing strain. In our data we detected a downregulation of involved proteins of cell wall synthesis but a slight upregulation of cell wall modification proteins. Moreover, we could not observe a significant change of expression of proteins responsible for a general stress response. Nonetheless, we chose the best possible system for heterologous expression of *Sa*NsrFP to overcome possible bottlenecks.

We show evidence that cells expressing *Sa*NsrFP obtain a modified cell wall: instead of an apartate/asparagine bridge in the pentapeptide found for the sensitive mutant, a species with two alanines was detected. In *Sa*NsrF_H202A_P, a mixture was found, although the two alanine species were present in only minor amounts (Fig. 7). This suggests that the transporter might already sense and mediate a second line of defense ATP-independently. ABC transporters that confer resistance against cationic antimicrobials are hypothesized to be involved or mediate modification processes of peptidoglycan in Gram-positive bacteria. d-alanylation of teichoic acids is assumed to diminish electrostatic attraction based on the observation that a lack of alanylation leads to increased binding to several positively charged molecules, e.g., gallidermin and vancomycin^41^. Additionally, the upregulation of the *gal* operon, especially of UDP-glucose-4-epimerase (GalE), influences the lipoteichoic acid (LTA) structure. GalE is responsible for the synthesis of $\alpha$-galactose, which is transported across the membrane to become a part of LTA^68^.

We can see this in our data with the upregulation of the MurQ which is responsible for the intracellular conversion of MurNAc-6P to *N*-acetylglucosamine-6-phosphate and d-lactate for the *Sa*NsrFP and *Sa*NsrF_H202A_P mutants in comparison to the sensitive strain. For transporter-expressing cells, we also observed an upregulation of proteins associated with antimicrobial resistance, such as UDP-glucose-4-epimerase and RodA^68^.

In the case that *Sa*NsrFP might mediate cell wall modifications upon receiving information on the cell wall targeting AMP, altered expression of genes could be the consequence. This finding might also reduce the number of proteins in the cytosol that are involved in lipid II biosynthesis, as seen by the whole proteome data where the expression of the genes is downregulated but not completely abolished. It needs to be verified whether *Sa*NsrFP is directly responsible for this or whether the *L. lactis* strain is reacting since its lipid II cycle is severely changed and, as a consequence, alters its cell wall composition.

Based on all results from this study, a joint activity of the transporter as a first-line defender and initiator for a second-line defense is very likely and results in resistance against compounds targeting the lipid II cycle and thus cell wall synthesis. By shielding the target UPP and lipid II from the extracellular space, e.g., by PGN modification that alters electrostatic attraction, less antibiotic, e.g., bacitracin, can be bound, and increased antibiotic concentrations can be detected in the supernatant. Our findings are in agreement with the previous conclusions for an export mechanism and further assumptions on the removal of AMPs from the membrane^34,44^. The tendency for upregulation of proteins associated with antimicrobial resistance and cell wall modification in *Sa*NsrFP-producing cell proteins indicates the activation of a second-line defense system.

Conclusively, BceAB-type transporters such as *Sa*NsrFP are evolutionarily conserved in human pathogenic and nonpathogenic strains. Although they are less conserved at the sequence level, the topology of the protein and their encoding operons are conserved. The resistance observed in different BceAB-type transporter studies indicates a common mechanism. The findings in this study are in line with a target protection mechanism, as was postulated for the BceAB transporter. Our data implies that AMP resistance is a far more complex process that involves a combination of an active target mechanism, which enables continuous growth, and a second line of defense, which could be initiated after sensing the AMP directly by the *Sa*NsrFP transporter.

## Materials and methods

### Cloning and expression

The plasmids pIL-SV SaNsrFP and pIL-SV SaNsrF_H202A_P, the latter harboring a point mutation in the H-loop, known to be crucial for ATP hydrolysis, were generated by cloning *nsrfp* from *S. agalactiae* COH1 as described in Alkhatib et al*.*^69^ and Reiners et al*.*^34^. Each plasmid and the empty vector pIL-SVCm was transformed into electrocompetent *L. lactis* NZ9000 cells^70^, and the resulting strains were termed NZ9000*Sa*NsrFP, NZ9000*Sa*NsrF_H202A_P and NZ9000Cm.

All strains used in this study have been described in previous publications^34,69^.

The *L. lactis* strains NZ9000*Sa*NsrFP and NZ9000*Sa*NsrF_H202A_P were cultured in GM17 medium containing 5–10 µg/ml chloramphenicol. Expression was induced by adding 0.3 nM nisin, and cultures were grown at 30 °C.

To analyze the expression, cultures were grown for 5 h and subsequently harvested using a centrifugation step for 30 min at 5000×*g*. The pellets were resuspended to an OD_600_ of 200 in resuspension buffer (50 mM HEPES pH 8.0, 150 mM NaCl, 10% glycerol), then 1/3 (w/v) 0.5 mm glass beads were added. The cells were lysed, and the supernatant was separated from cell debris as well as glass beads by centrifuging at 10,000×*g*. Subsequently, the membranes were harvested from the supernatant by a 100,000×*g* centrifugation step. Membrane fractions were mixed with SDS-loading dye (0.2 M Tris–HCl, pH 6.8, 10% (w/v) SDS, 40% (v/v) glycerol, 0.02% (w/v) bromophenol and β-mercaptoethanol) and used for SDS-PAGE and western blot analysis. A polyclonal antibody against the extracellular domain of *Sa*NsrP was used to detect the expressed *Sa*NsrFP protein (Davids Biotechnologie, Regensburg, Germany).

### Biological assays

#### Purification of nisin

Nisin was purified with ion-exchange chromatography as previously described^71^, and the concentration was determined with RP-HPLC according to Abts et al.^72^.

#### Determination of the half-maximal inhibitory concentration (IC_50_)

The half maximal inhibitory concentration was determined according to Abts et al.^71^. Briefly, *L. lactis* NZ9000Cm, *L. lactis* NZ9000*Sa*NsrFP and *L. lactis* NZ9000*Sa*NsrF_H202A_P cells were grown in GM17 medium containing 5 µg/ml chloramphenicol and 0.3 nM nisin at 30 °C overnight. Fresh GM17Cm medium with a sublethal amount of nisin (0.3 nM) was inoculated with overnight cultures to an OD_600_ of 0.1. A 96-well plate was prepared with a serial dilution of examined antibiotics (concentration ranges ramoplanin 0.014 nM–3.75 µM; lysobactin 0.002 nM–10 µM; vancomycin 0.02 nM–80 µM; nisin 0.0001 nM–0.5 µM) and subsequently the cell culture was added and plates were incubated at 30 °C for 5 h. Afterwards, the optical density was measured, and the IC_50_ values for each strain and antibiotic were calculated^34^. To make those values more comparable, the fold of resistance was determined by dividing the IC_50_ values of *L. lactis* NZ9000*Sa*NsrFP and *L. lactis* NZ9000*Sa*NsrF_H202A_P by the corresponding value for *L. lactis* NZ9000Cm.

#### Growth curves

To detect the growth behavior of the different strains, precultures of *L. lactis* NZ9000Cm, *L. lactis* NZ9000*Sa*NsrFP and *L. lactis* NZ9000*Sa*NsrF_H202A_P cells were grown in GM17 medium with 5 or 10 µg/ml chloramphenicol and 0.3 nM nisin at 30 °C overnight. Freshly prepared GM17Cm medium with 0.3 nM nisin was inoculated with overnight cultures to an OD_600_ of 0.1 and grown to an OD_600_ of 0.4–0.5 at 30 °C. Afterwards, the cells were diluted to an OD_600_ of 0.05 in GM17Cm medium containing 0.3 nM nisin. Cells were treated with either 1 µM bacitracin and 1 mM ZnCl_2_ or 4 µM bacitracin without ZnCl_2_. Growth was detected at OD_584_ every 10 min with a FLUOstar OPTIMA (BMG Lab technology).

### Cell wall precursor analysis

#### Growth condition and sample preparation

Cells were grown in M17 medium supplemented with 0.5% glucose and 0.3 nM nisin overnight at 30 °C without shaking. The next day, 100 ml with 0.5% glucose and 0.3 nM nisin was inoculated with overnight cultures to OD_600_ = 0.1. When OD_600_ = 1.2 bacitracin (100 µg/ml) was added to the cultures to enrich cell wall precursors, and the cultures were incubated for an additional 30 min at 30 °C. This step was repeated once. (As a control, a second culture each was harvested before bacitracin was added at an OD_600_ = 1.2, and cell pellets were stored at − 20 °C) After incubation with bacitracin, the cells were harvested, and the cell pellets were stored at − 20 °C. The next day, the cell pellets were resuspended in 25 ml water and cooked for 60 min in boiling water to break the cells. Cell debris was removed by centrifugation (15 min, 500×*g*, 4 °C). The supernatant, containing the cell wall precursors, was lyophilized overnight. Cell pellets were resuspended in 150 µl water and used for LC/MS analysis.

### LC/MS analysis of cell wall fragments

Five microliters of each sample were injected into an XCT6330 LC/MSD ultratrap system (Agilent Technologies) equipped with a Nucleosil 100 C18 column (3 μm × 100 mm × 2 mm internal diameter, Dr. Maisch GmbH). The column was used at 40 °C. A linear gradient was performed from 0 to 10% eluent B (0.06% formic acid in acetonitrile) over 25 min with a flow rate of 400 µl/min. The column was re-equilibrated for 10 min with 100% buffer A (0,1% formic acid in water). Ionization alternated between positive and negative ion modes with a capillary voltage of 3.5 kV at 350 °C. Extracted ion chromatograms (EICs) in negative ion mode for UDP-MurNAc-l-Ala-d-iGlu-l-Lys-d-Ala-d-Ala (m/z^−1^ 1148.34 ± 0.1) and UDP-MurNAc-l-Ala-d-iGlu-l-Lys-(d-Asp)-d-Ala-d-Ala (m/z^−1^ 1263.37 ± 0.1) were analyzed with Data Analysis (Bruker), exported and presented with GraphPad Prism 6.0.

### Peptidoglycan analysis

#### Isolation of peptidoglycan

600 ml main culture of *L. lactis* NZ9000Cm, *L. lactis Sa*NsrFP and *L. lactis Sa*NsrF_H202A_P were inoculated with overnight culture and incubated to an OD600 of 0.1 at 30 °C. After reaching the late exponential growth phase, the cells were harvested. To isolate the peptidoglycan, the cells were thawed on ice and resuspended in 15 ml of 50 mM Tris/HCl buffer pH 7.0. The cell suspension was added dropwise to 60 ml of boiling, stirred 4% SDS solution. After boiling for another 15 min, the suspension was cooled to room temperature and centrifuged at 13,000×*g* for 10 min. The pellet was washed twice with 1 M NaCl followed by water until no SDS was detectable in the supernatant. Pellet was resuspended in 1 ml water, and 1/3 volume of glass beads (Æ 0.5 mm) were added. After cell lysis the glass beads were harvested at 2000×*g* for 5 min. The supernatant was centrifuged at 25,000×*g* for 15 min, and the pellet containing the cell walls were resuspended in 100 mM Tris/HCl pH 8.5 buffer with 20 mM MgSO_4_. After addition of 10 µg/ml DNase I and 50 µg/ml RNase, the samples were incubated at 37 °C with 180 rpm for 2 h. Following the addition of 10 mM CaCl_2_ and 100 µg/ml trypsin, an 18 h incubation was performed under the same conditions. Enzymatic activities were stopped by the addition of 1% SDS and incubation at 80 °C for 15 min. The suspension was diluted to 20 ml with water and centrifuged at 25,000×*g* for 30 min. The pellet was resuspended and incubated at 37 °C for 15 min with 10 ml 8 M LiCl and 10 ml 100 mM EDTA pH7, respectively. The peptidoglycan pellet was washed with water, acetone and water and was lyophilized.

Samples were treated as follows: 150 µl of resuspended peptidoglycan were mixed with 60 µl of mQ water and with 75 µl of TES buffer (200 mM TES, 4 mM MgCl_2_, pH 7.0 with final concentration in sample: 150 mM TES, 3 mM MgCl_2_, pH 7.0) and 15 µl of mutanolysine (75U) (Sigma-Aldrich, 5 kU/ml, dissolved in mQ water). Samples were incubated overnight at 37 °C and then centrifuged at RT for 5 min at 14,000 rpm. 90 µl of the supernatant were used for HPLC–MS analysis.

#### HPLC–MS analysis of muropeptides

90 µl of the sample were injected for HPLC–MS analyses (XCT 6330 LC/MSD Ultra Trap system; Agilent Technologies) and Reprosil-Gold 300 C_18_ column (5 µm by 250 mm by 4.6 mm internal diameter). The HPLC parameters were as follows: Holding with 5% of solvent B (methanol + 0.06% HCOOH) for 5 min and then start with a linear gradient from 30% solvent B to 70% solvent A (water + 0.1% HCOOH) for 150 min with additional holding with 30% solvent B over 30 min at a flow rate of 500 µl/min. The MS parameters were as follows: Ionization alternating positive and negative, capillary voltage 3.5 kV, and temperature 350 °C.

### Proteome analysis

#### Sample preparation

The *L. lactis* strains NZ9000*Sa*NsrFP and NZ9000*Sa*NsrF_H202A_P were grown at 30 °C in GM17 medium containing 5 µg/ml chloramphenicol and 0.3 nM nisin. Precultures were inoculated to an OD_600_ of 0.1 and grown to the exponential growth phase before a main culture was inoculated to an OD_600_ of 0.1. The cells were harvested using 5000×*g*, and the pellets were resuspended in phosphate buffer pH 7 to an OD_600_ of 200. Then, 1/3 (w/v) 0.5 mm glass beads were added. The cells were lysed, and the supernatant was separated by centrifugation at 10,000×*g*.

Protein concentration was determined by means of a Pierce 660 nm Protein Assay (Fischer Scientific, Schwerte, Germany), and 10 µg protein per sample was loaded on an SDS-PAGE gel for in-gel digestion. The isolated gel pieces were reduced, alkylated and underwent tryptic digestion. The peptides were resolved in 0.1% trifluoracetic acid and subjected to liquid chromatography.

### LC–MS analysis

For the LC–MS analysis, a QExactive plus (Thermo Scientific, Bremen, Germany) connected with an Ultimate 3000 Rapid Separation liquid chromatography system (Dionex/Thermo Scientific, Idstein, Germany) equipped with an Acclaim PepMap 100 C18 column (75 µm inner diameter, 25 cm length, 2 mm particle size from Thermo Scientific, Bremen, Germany) was applied. The length of the LC gradient was 120 min. The mass spectrometer was operated in positive mode and coupled with a nano electrospray ionization source. The capillary temperature was set to 250 °C, and the source voltage was set to 1.4 kV. In the QExactive plus mass spectrometer for the survey scans, a mass range from 200 to 2000 m/z at a resolution of 70,000 was used. The automatic gain control was set to 3,000,000, and the maximum fill time was 50 ms. The 10 most intensive peptide ions were isolated and fragmented by high-energy collision dissociation (HCD).

### Computational mass spectrometric data analysis

Proteome Discoverer (version 2.1.0.81, Thermo Fisher Scientific, Bremen, Germany) was applied for peptide/protein identification by applying Mascot (version 2.4, Matrix Science, London, UK) as a search engine employing the EnsemblBacteria database (*Lactococcus lactis* subsp. *cremoris* NZ9000; date 03-11-2019). A false discovery rate of 1% (p ≤ 0.01) at the peptide level was set as the identification threshold. Proteins were quantified with Progenesis QI for Proteomics (Version 2.0, Nonlinear Dynamics, Waters Corporation, Newcastle upon Tyne, UK). Only proteins containing at least two unique peptides were taken into consideration. For the calculation of enriched proteins in the groups, a 5% false discovery rate and a minimum fold change of two were used.

The mass spectrometry proteomics data have been deposited to the ProteomeXchange Consortium via the PRIDE partner repository with the data set identifier PXD017318.

The protein lists, which have been uploaded to PRIDE, are also provided as Supplementary Material.

## Supplementary Information

Supplementary Information.

## Abbreviations

UDP: Undecaprenyl-phosphate
GlcNAc: *N*-acetylglucosamine
MurNAc: *N*-acetylmuramic acid
MurN: *N*-deacetylated muramic acid
Ala: Alanine
iGlu: Isoglutamic acid
iGln: Isoglutamine
Glu: Glutamine
Asp: Aspartate/aspartic acid
Asn: Asparagine
Lys: Lysine
