# Supplementary material for: New insights into the resistance mechanism for the BceAB-type transporter SaNsrFP
Source: Sci Rep. 2022 Mar 10;12:4232. doi: 10.1038/s41598-022-08095-2 (PMC8913810; doi:10.1038/s41598-022-08095-2)
Supplement: Supplementary file 1 — Supplementary Information. [file 41598_2022_8095_MOESM1_ESM.pdf]

New insights into the resistance mechanism for the BceAB-type transporter *Sa*NsrFP

Julia Gottstein[a*], Julia Zaschke-Kriesche[a*], Sandra Unsleber[b], Irina Voitsekhovskaia[b], Andreas Kulik[b], Lara V. Behrmann[a], Nina Overbeck[c], Kai Stühler[c], Evi Stegmann[b] and Sander H.J. Smits[a*]

[a] Institute of Biochemistry, Heinrich-Heine-University Duesseldorf, Universitaetsstrasse 1, 40225 Duesseldorf, Germany.

[b] Interfaculty Institute of Microbiology and Infection Medicin, Eberhard Karls University, Auf der Morgenstelle 28, 72076 Tübingen, Germany.

[c] Molecular Proteomics Laboratory, Heinrich-Heine-University Duesseldorf, Universitaetsstrasse 1, 40225 Duesseldorf, Germany.

*Corresponding author: Sander H. J. Smits, Institute of Biochemistry, Heinrich-Heine-University Duesseldorf, Universitaetsstrasse 1, 40225 Duesseldorf, Germany.

* both authors contributed equally

Email: Sander.Smits@hhu.de

SI Figure 1

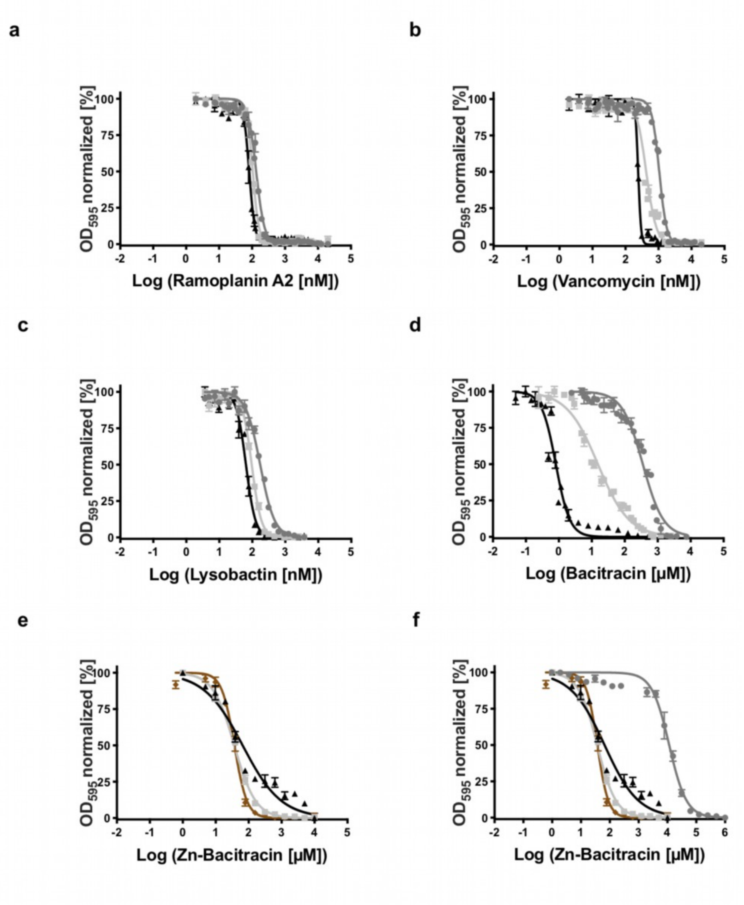

SI Figure 1: Representative inhibitional growth curves of **a) Ramoplanin A2**, **b) Vancomycin**, **c) Lysobactin, d) Bacitracin e) & f) Zn-Bacitracin.** The normalized OD_{595} is plotted against the logarithmic concentration of the antibiotic. NZ9000Cm is demonstrated in black, NZ9000*Sa*NsrF_{H202A}P in light grey, NZ9000*Sa*NsrFP in grey and NZ9000NisT in brown.

SI Figure 2.

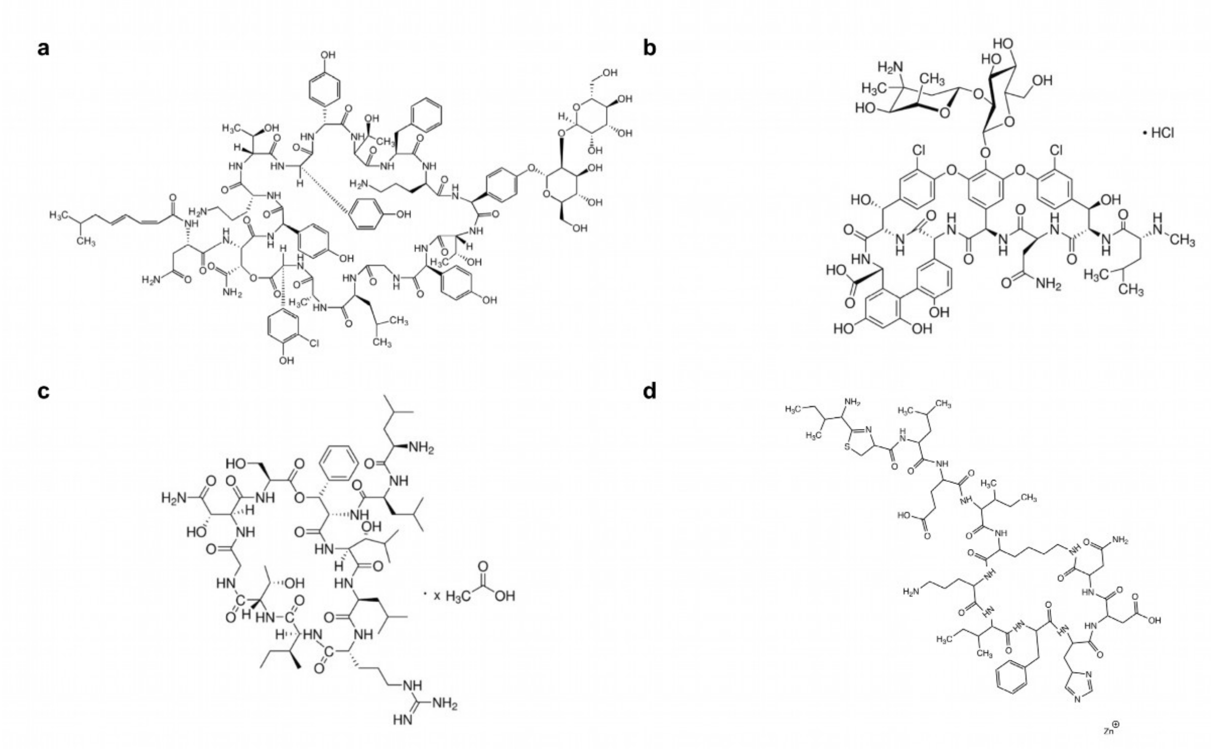

SI Figure 2: Structures of a) Ramoplanin A2 from AdipoGen life sciences, **b) Vancomycin** from Fluka Analytical, **c) Lysobactin** from Sigma life sciences and **d) Bacitracin** from Fisher BioReagents.

ADJ60406

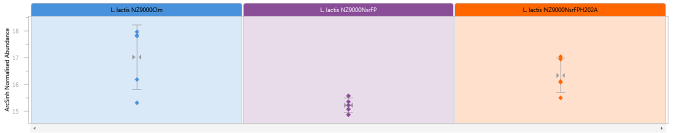

ADJ60417

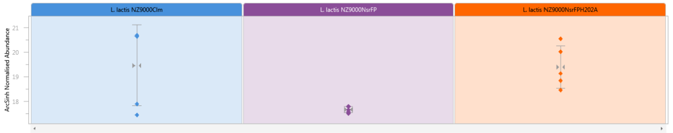

ADJ60503

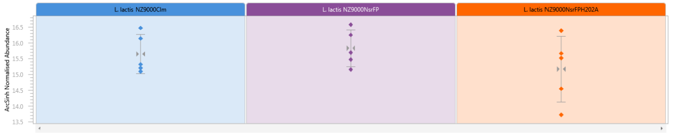

ADJ60765

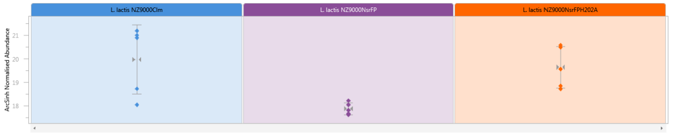

ADJ60806

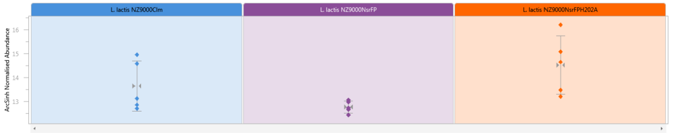

ADJ60966

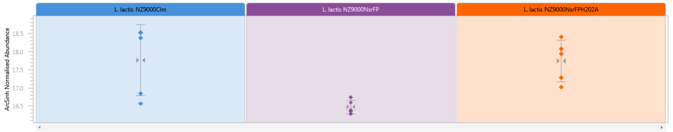

ADJ59149

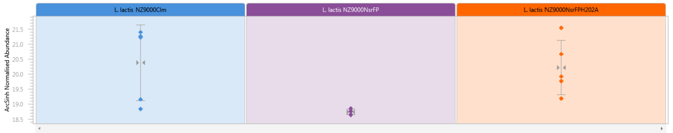

ADJ59249

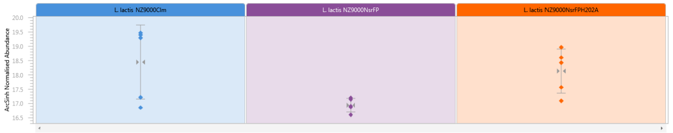

ADJ59347

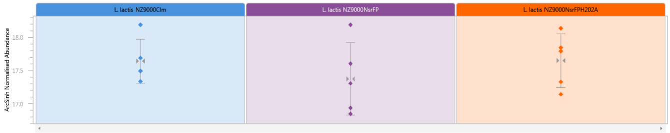

ADJ59379

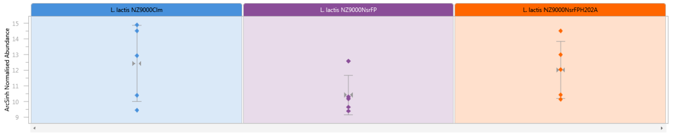

ADJ59381

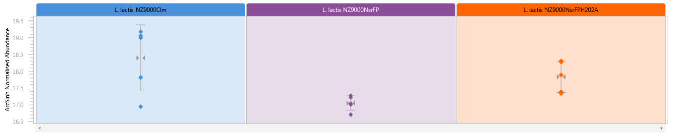

ADJ59382

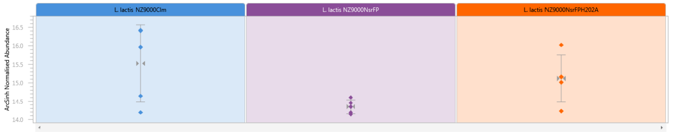

ADJ60973

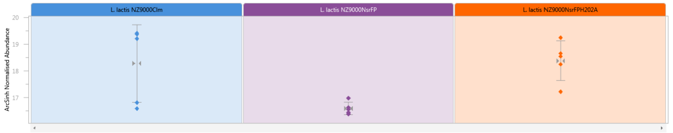

ADJ61030

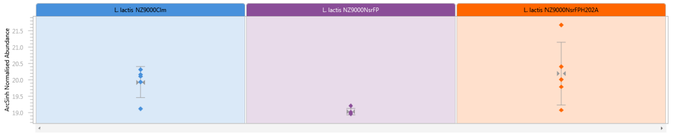

ADJ61045

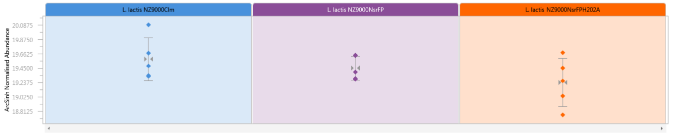

ADJ61135

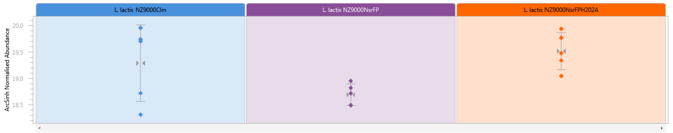

ADJ61146

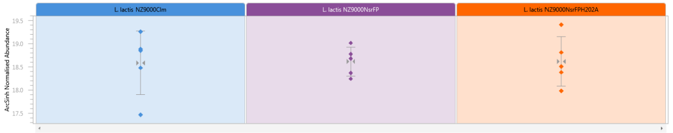

ADJ61268

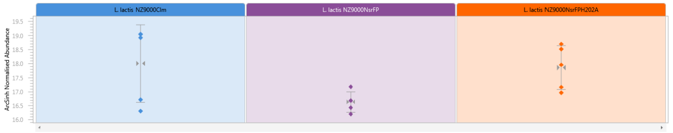

ADJ61283

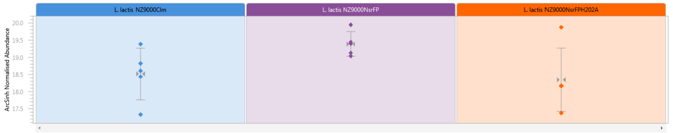

ADJ61304

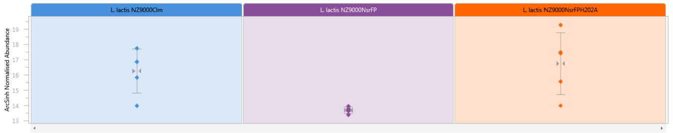

ADJ61321

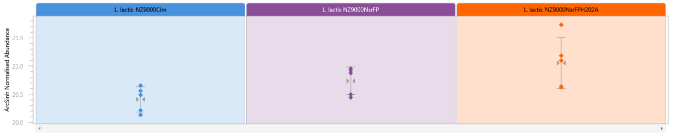

ADJ61415

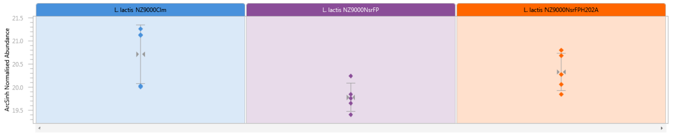

ADJ61465

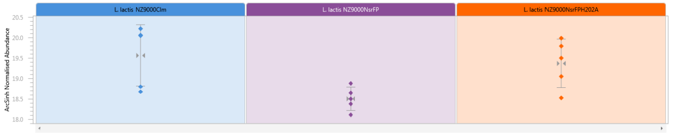

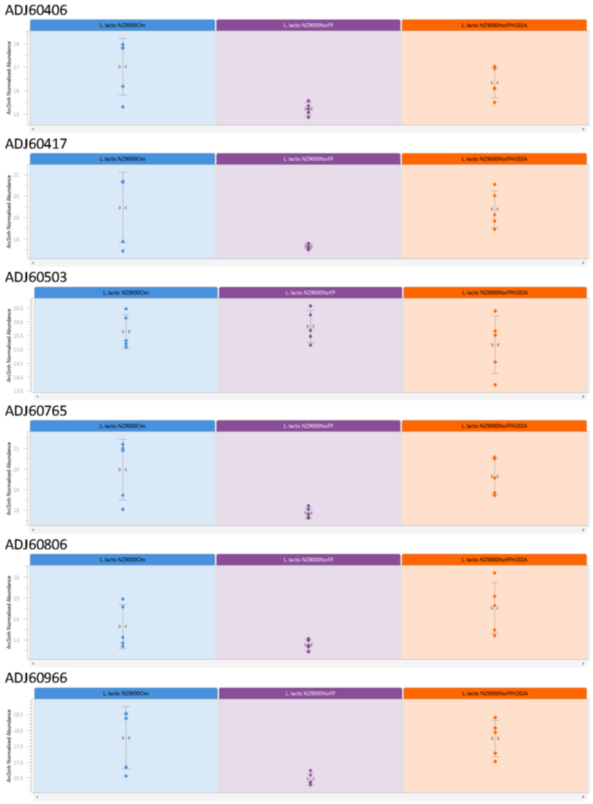

SI Figure 3: Normalized abundance of several proteins of the proteome analysis of *L. lactis* NZ9000Cm (blue), NZ9000NsrFP (violette) and NZ9000NsrF$_{H202A}$P (orange).

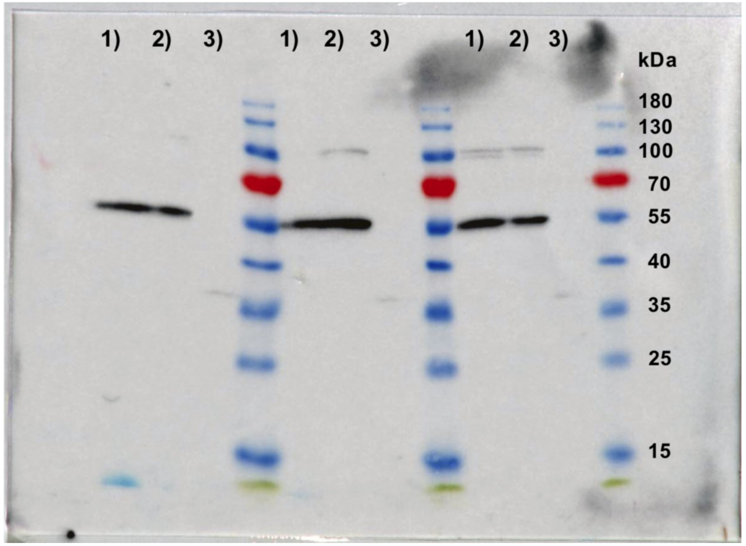

SI Figure 4: Expression of *Sa*NsrFP (1) and *Sa*NsrF$_{H202A}$P (2) and the empty vector pIL-SV (3) in *L. lactis* NZ9000Cm from three different cultures, monitored *via* western blot with a polyclonal antibody against the extracellular domain of *Sa*NsrP**.**

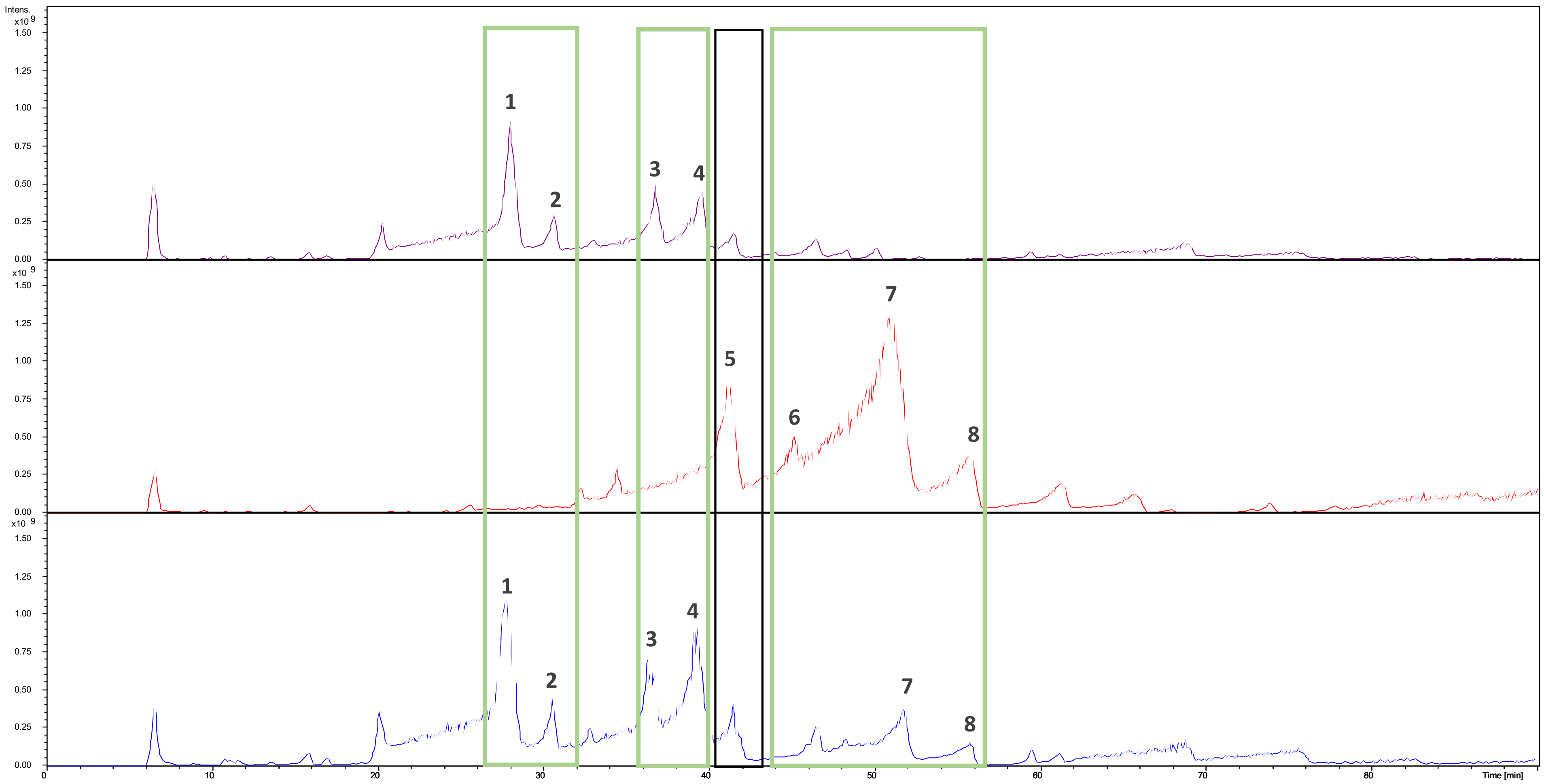

SI Figure 5: LC-MS Chromatogram of isolated muropeptides from *L. lactis* NZ9000Cm (purple), *L. lactis* NZ9000NsrFP (red) and *L. lactis* NZ9000NsrF$_{H202A}$P (blue). Marked peaks in green frames could be related to the mass of GlcNAc-MurNAc-L-Ala-D-iGln-L-Lys-(D-Asn) (peak 1), GlcNAc-MurNAc-L-Ala-D-iGln-L-Lys-(D-Asp) (peak 2), GlcNAc-MurNAc-L-Ala-D-iGln-L-Lys-(D-Asn)-D-Ala (peak 3), GlcNAc-MurNAc-L-Ala-D-iGln-L-Lys-(D-Asn)-D-Ala-D-Ala (peak 4) and GlcNAc-MurNAc-L-Ala-D-iGln-L-Lys-(Ala-Ala)-D-Ala-D-Ala (peaks 6-8). Marked peak 5 in black frame could be related to the mass GlcNac-MurNac-L-Ala-D-iGln-L-Lys-(Ala)-D-Ala-D-Ala.

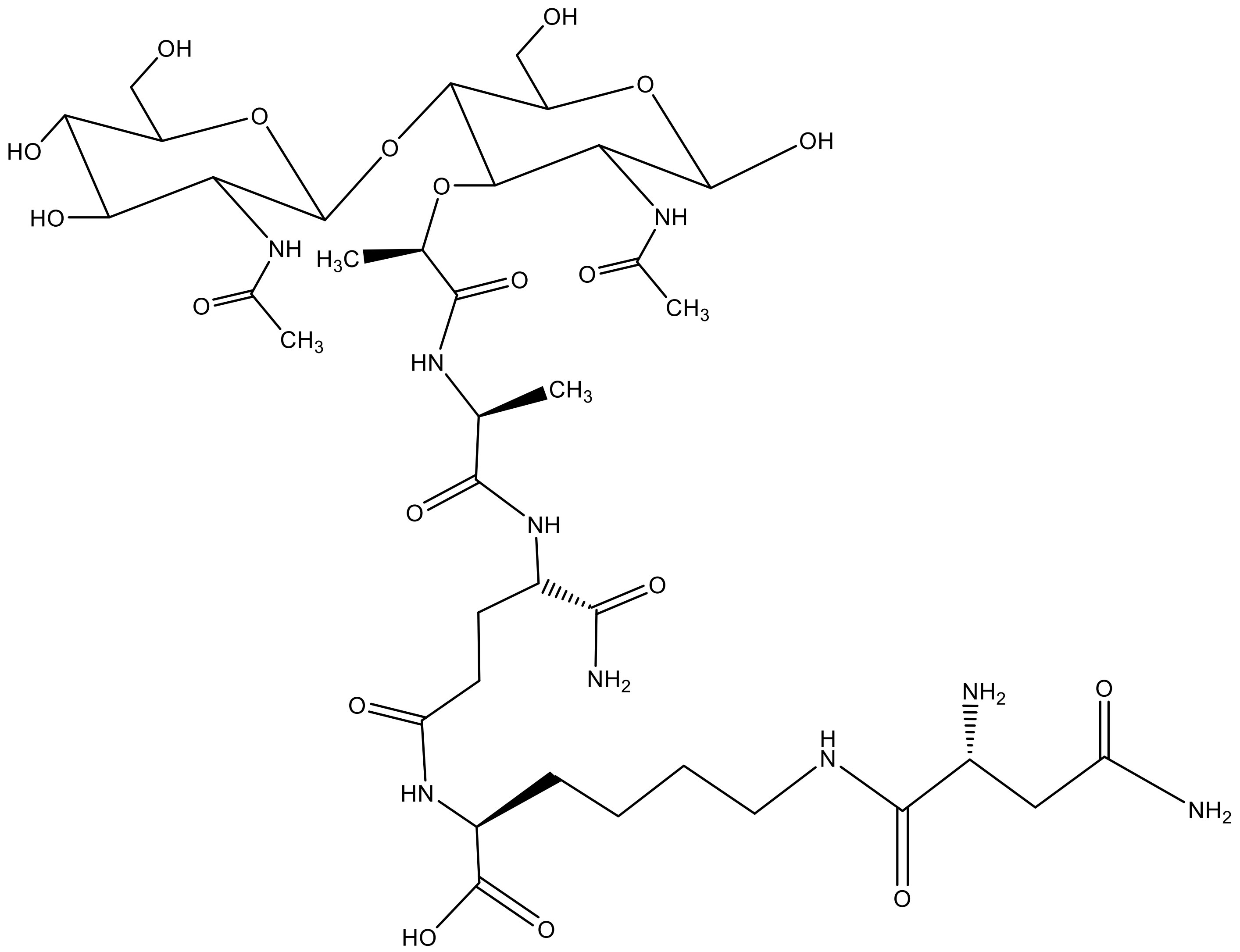

SI Figure 5a: Muropeptide structure corresponding to peak 1.

GlcNAc-MurNAc-L-Ala-D-iGln-L-Lys-(D-Asn) detected in *L. lactis* NZ9000Cm and *L. lactis* NZ9000*Sa*NsrF$_{H202A}$P with *m/z* 938.37 [M+H]$^{+}$.

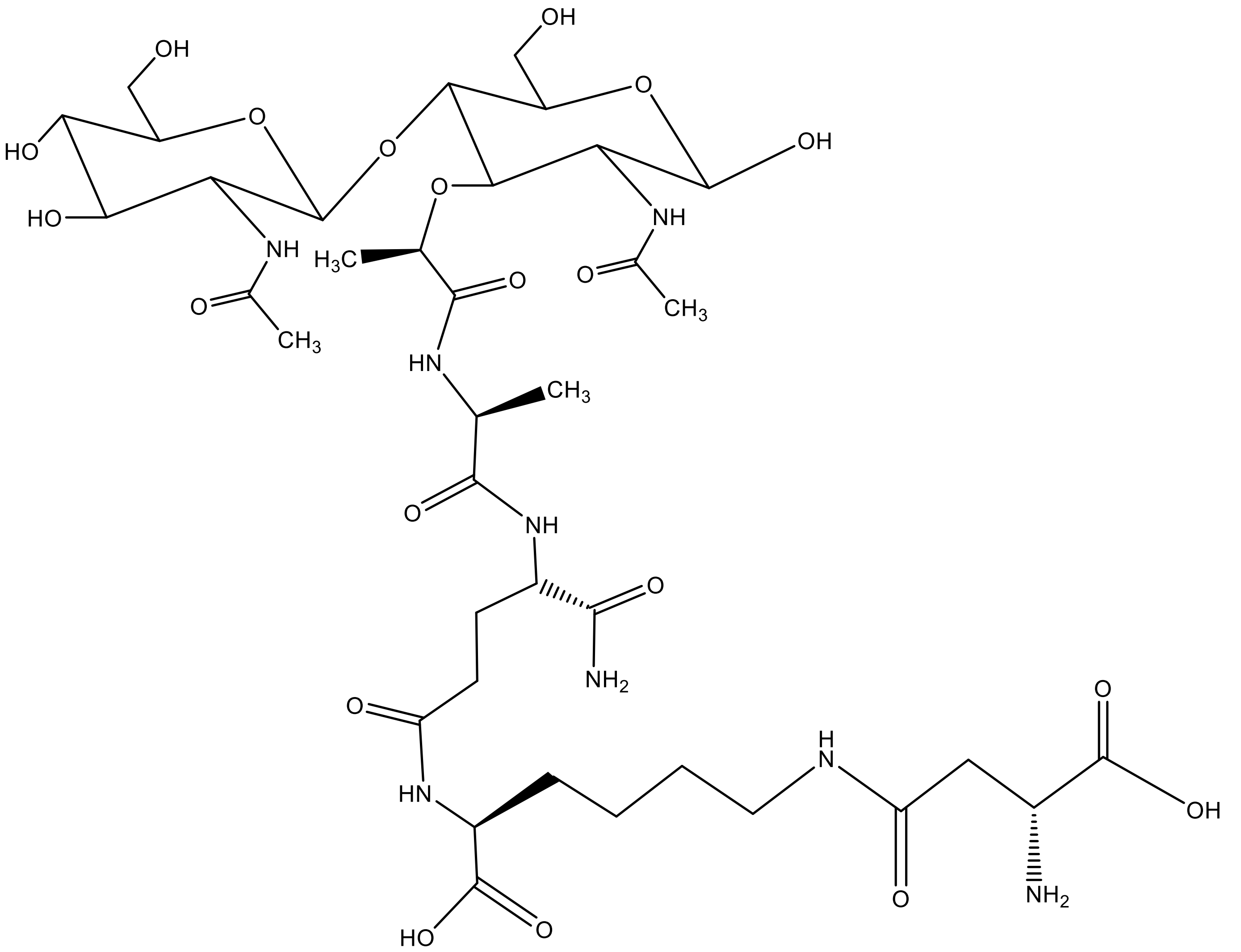

SI Figure 5b: Muropeptide structure corresponding to peak 2.

GlcNAc-MurNAc-L-Ala-D-iGln-L-Lys-(D-Asp) detected in *L. lactis* NZ9000Cm and *L. lactis* NZ9000*Sa*NsrF$_{H202A}$P with *m/z* 939.37 $[M+H]^+$.

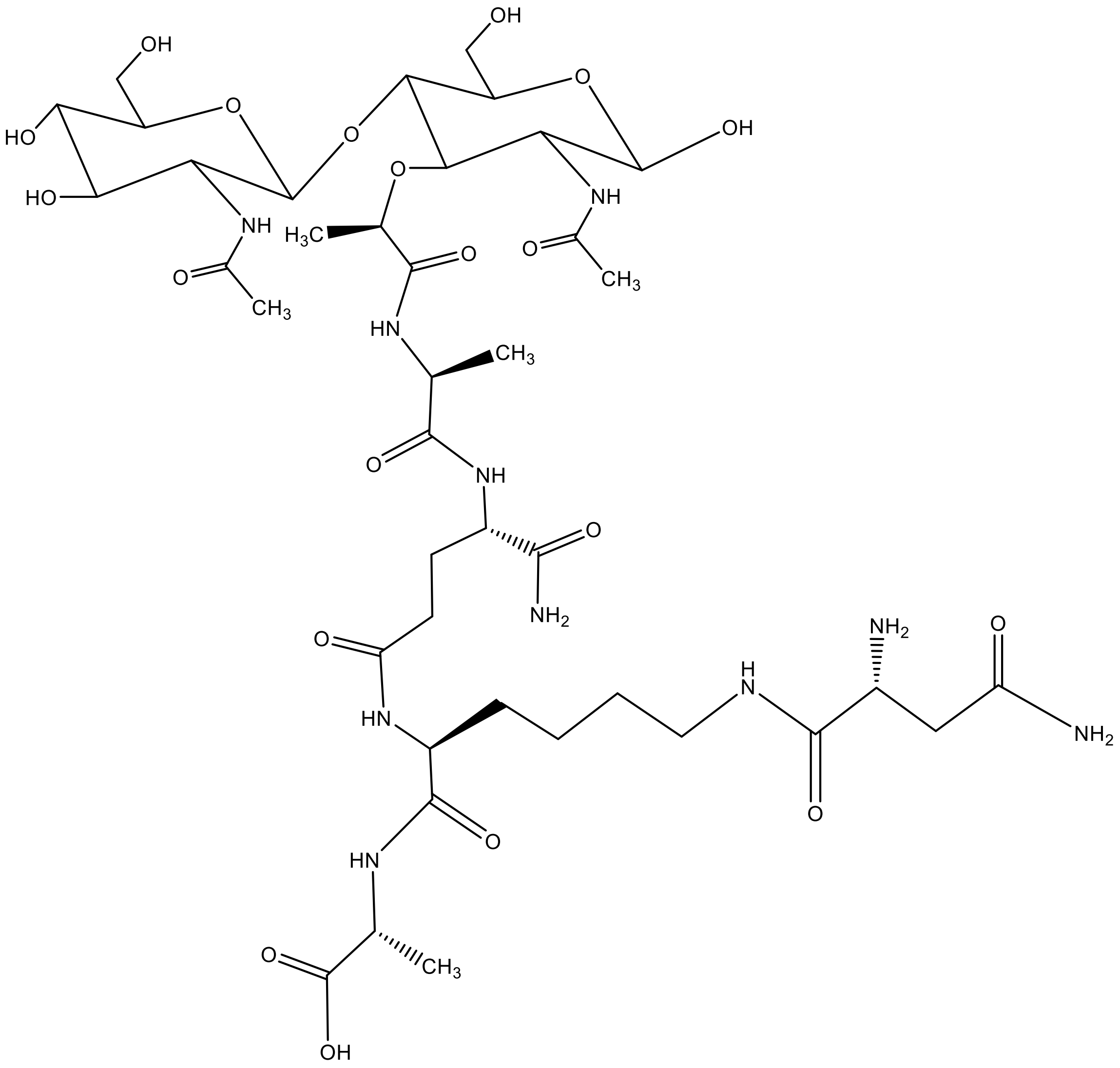

SI Figure 5c: Muropeptide structure corresponding to peak 3.

GlcNAc-MurNAc-L-Ala-D-iGln-L-Lys-(D-Asn)-D-Ala detected in *L. lactis* NZ9000Cm and *L. lactis* NZ9000*Sa*NsrF$_{H202A}$P with *m/z* 1009.45 $[M+H]^+$.

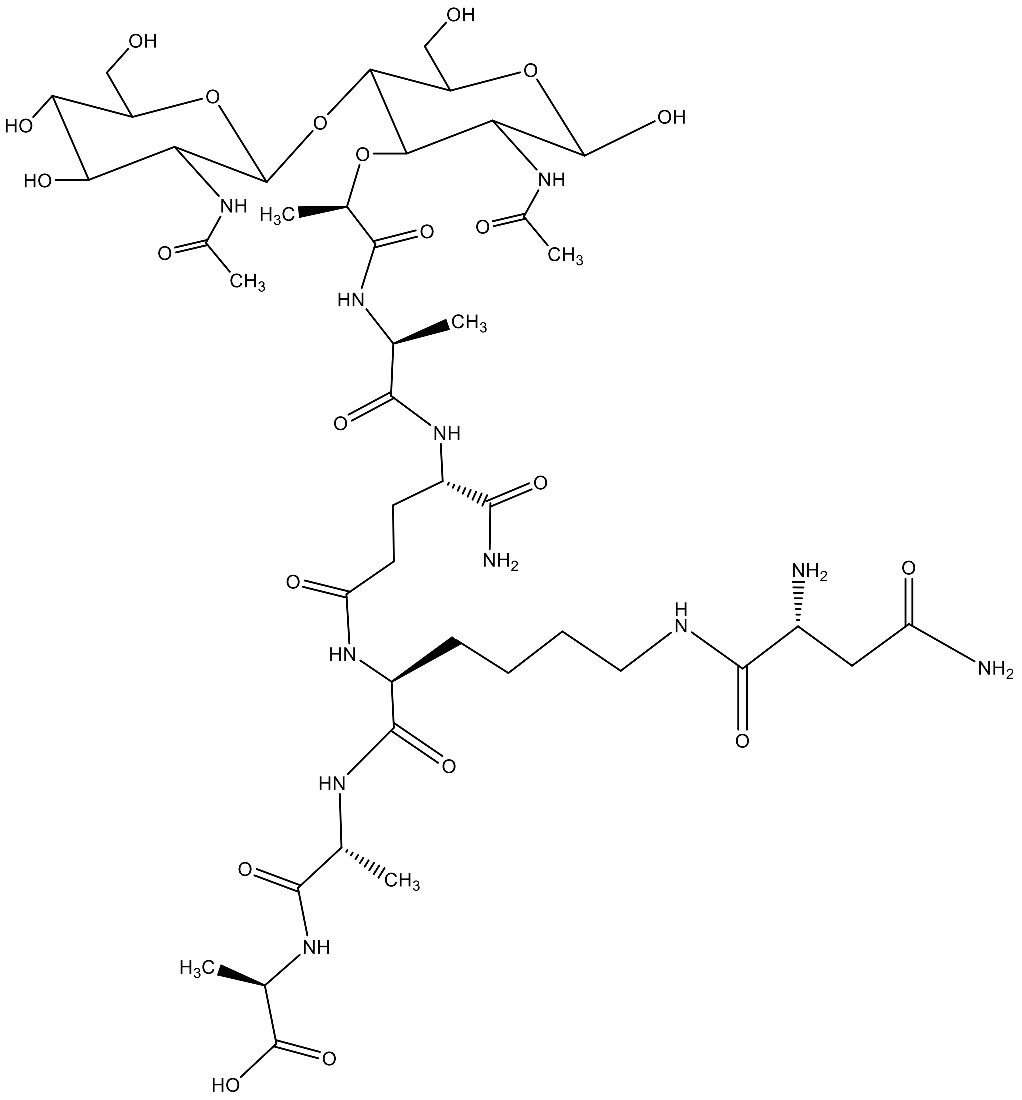

SI Figure 5d: Muropeptide structure corresponding to peak 4.

GlcNAc-MurNAc-L-Ala-D-iGln-L-Lys-(D-Asn)-D-Ala-D-Ala detected in *L. lactis* NZ9000Cm and *L. lactis* NZ9000*Sa*NsrF$_{H202A}$ with *m/z* 1080.50 $[M+H]^+$.

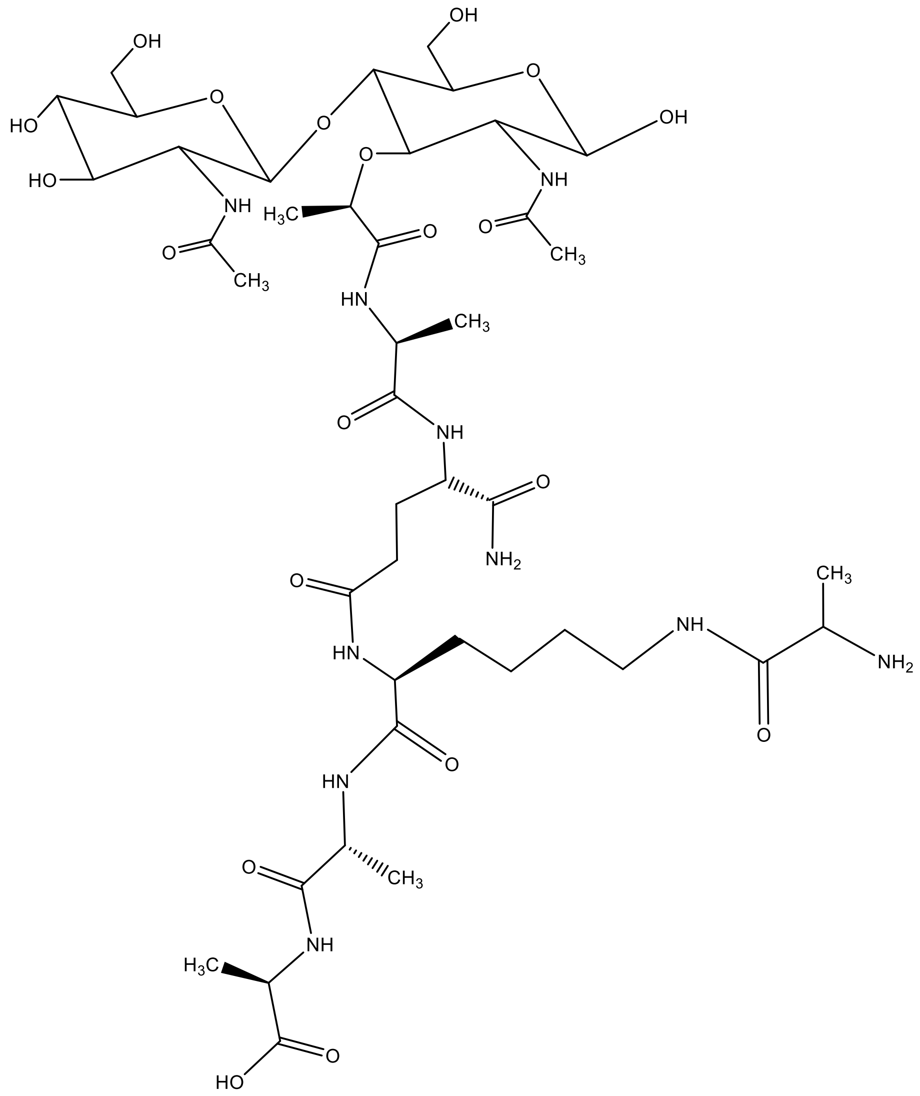

SI Figure 5e: Muropeptide structure corresponding to peak 5.

GlcNAc-MurNAc-L-Ala-D-iGln-L-Lys-(Ala)-D-Ala-D-Ala detected in *L. lactis* NZ9000*Sa*NsrFP with *m/z* 1037.49 $[M+H]^+$.

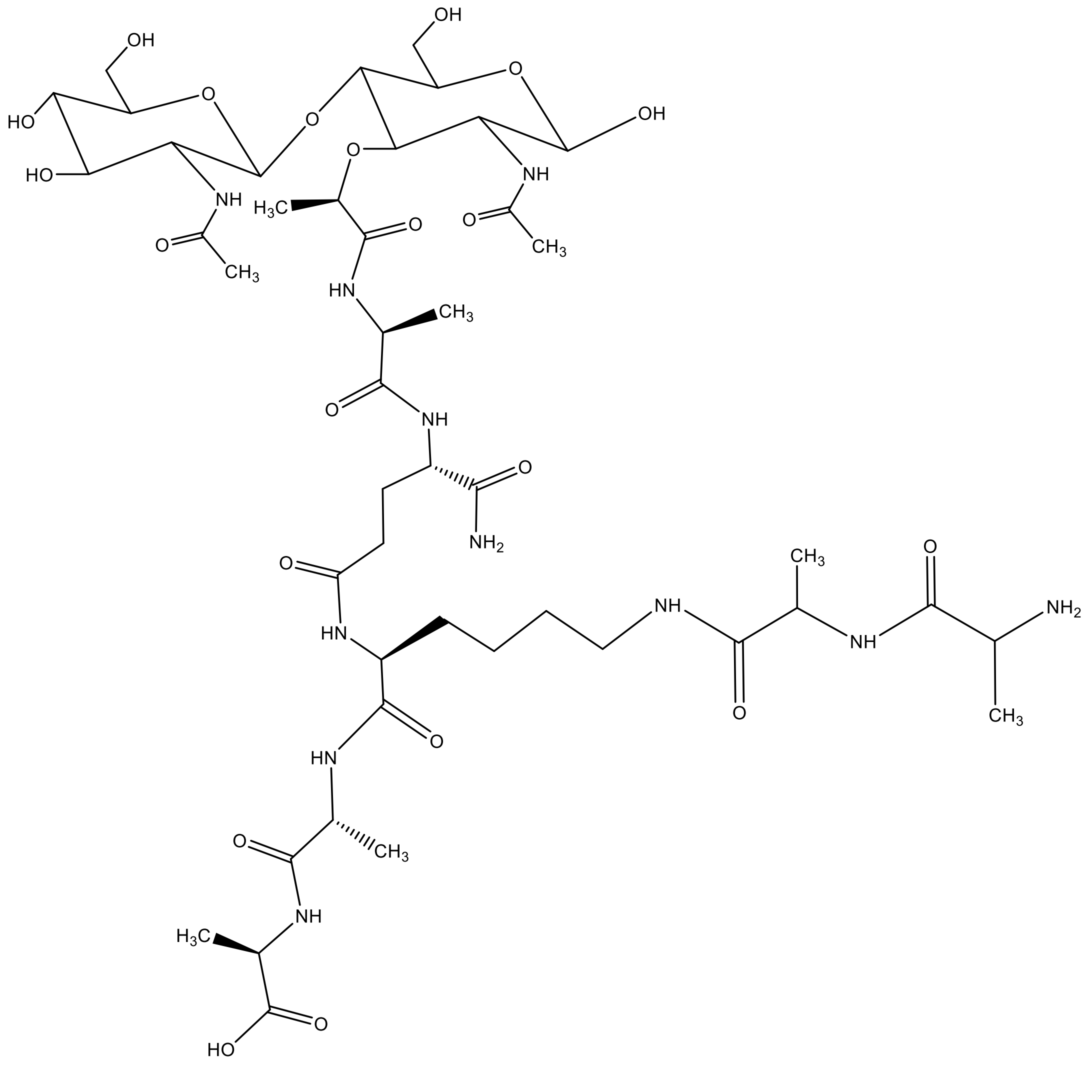

SI Figure 5f: Muropeptide structure corresponding to peaks 6-8.

GlcNAc-MurNAc-L-Ala-D-iGln-L-Lys-(Ala-Ala)-D-Ala-D-Ala detected in *L. lactis* NZ9000*Sa*NsrFP and *L. lactis* NZ9000*Sa*NsrF$_{H202A}$P with *m/z* 1108.53 $[M+H]^+$.

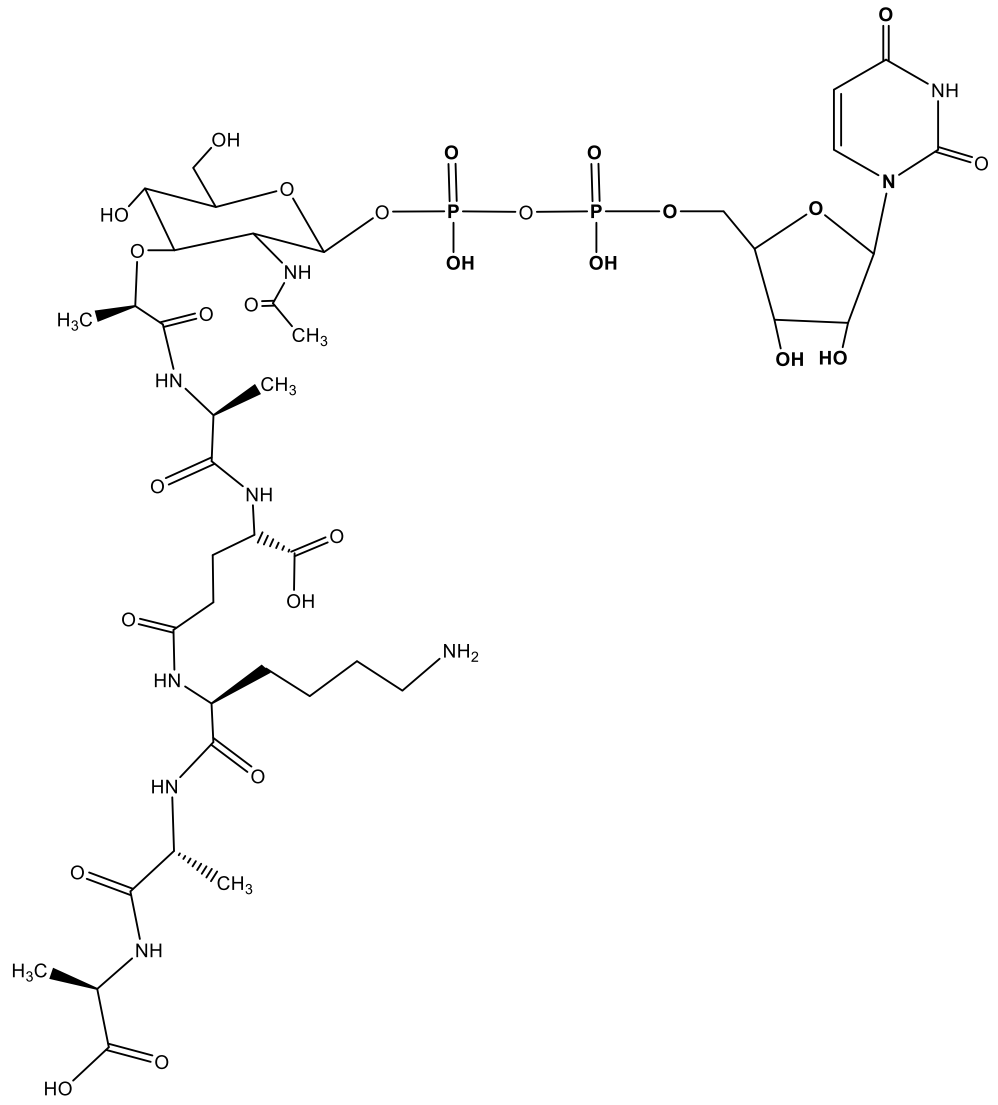

SI Figure 6a: UDP-linked intermediate detected in this study.

UDP-MurNAc-L-Ala-D-iGlu-L-Lys-D-Ala-D-Ala detected in *L. lactis* NZ9000*Sa*NsrF$_{H202A}$P and *L. lactis* NZ9000*Sa*NsrFP with mass m/z-1 (1148.4).

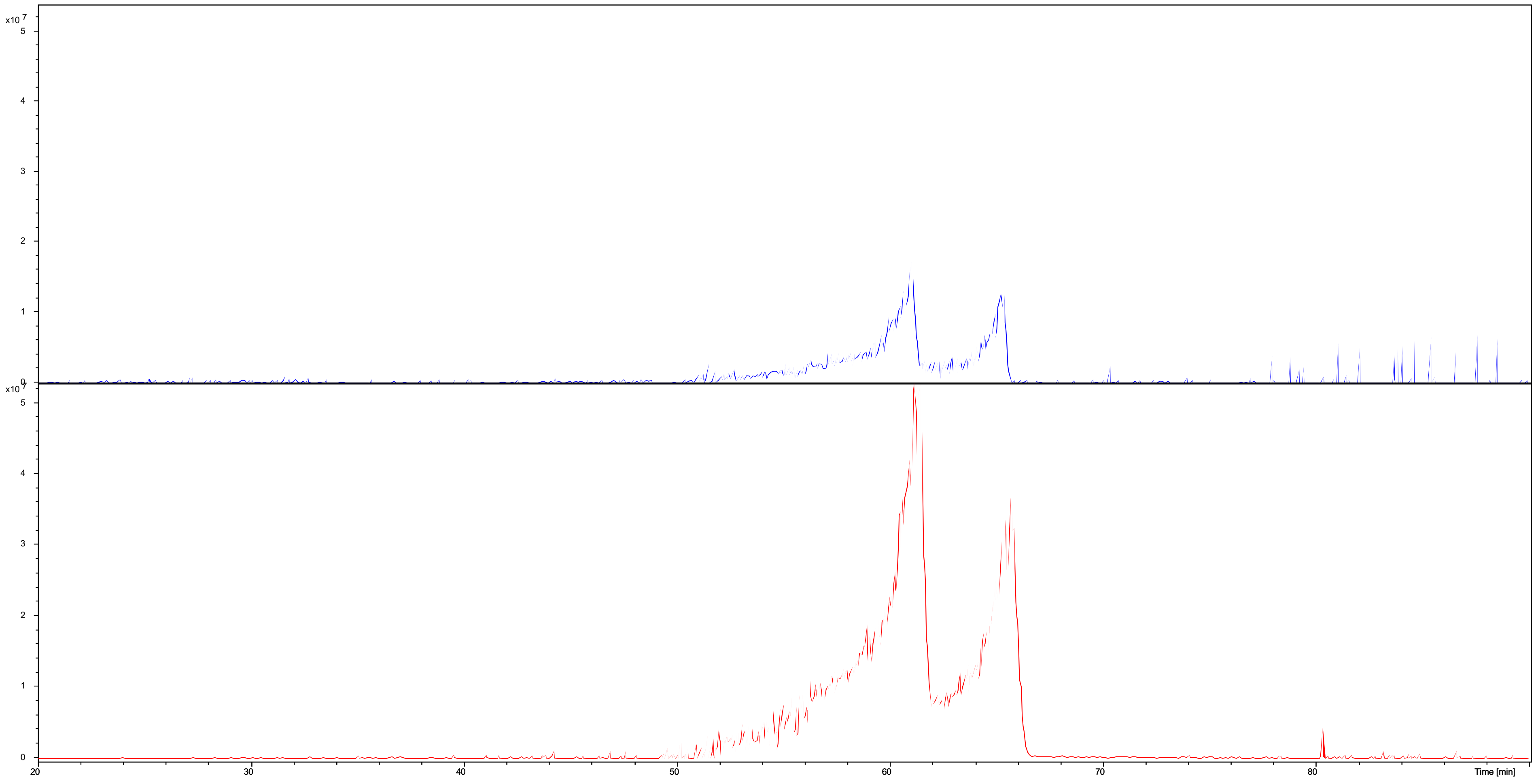

SI Figure 6b: Extracted ion chromatography (EIC) spectrum of UDP-linked intermediate detected in this study.

(EIC) spectrum of UDP-MurNAc-L-Ala-D-iGlu-L-Lys-D-Ala-D-Ala detected in *L. lactis* NZ9000*Sa*NsrF$_{H202A}$P (blue) *L. lactis* NZ9000*Sa*NsrFP (red) with mass m/z-1 (1148.4).

SI Table 1: A two-sided Students t-test was performed using Graphpad Prism version 9.2.0 with the IC_{50} data obtained for *Sa*NsrFP and *Sa*NsrF$_{H202A}$P. P-values were listed. A two-sided, unpaired Students t-test was performed using Graphpad Prism version 9.2.0 for Windows, GraphPad Software, San Diego, California USA, www.graphpad.com".

Antibiotic	p-values ($p<0.05$)
**Ramoplanin A2**	ns
**Vancomycin**	0.0228
**Lysobactin**	0.0076
**Bacitracin**	0.0001
**Bacitracin $ZnCl_2$**	<0.0001
**Nisin**	<0.0001
**Gallidermin**	<0.0001
